# Spectroscopic Exploration of Squaraine Dyes: Molecular Characterization of Fundamental, Combination, and Overtone Bands

**DOI:** 10.1021/acsphyschemau.5c00079

**Published:** 2025-12-18

**Authors:** Edoardo Buttarazzi, Vittoria Burigana, Elisabetta Collini, Alessio Petrone

**Affiliations:** † 657489 Scuola Superiore Meridionale, Largo San Marcellino 10, I-80138 Napoli, Italy; ‡ Department of Chemical Sciences, 9308 University of Napoli Federico II, Complesso Universitario di Monte S. Angelo, via Cintia 21, I-80126 Napoli, Italy; § Department of Chemical Sciences, University of Padova, Via Marzolo 1, I-35131 Padova, Italy; ∥ Istituto Nazionale Di Fisica Nucleare, sezione di Napoli, 201813 Complesso Universitario di Monte S. Angelo ed. 6, Via Cintia, I-80126 Napoli, Italy

**Keywords:** spectroscopy, resonance Raman, vibrational analysis, anharmonic VPT2 formalism, squaraine dyes, infrared

## Abstract

A comprehensive vibrational analysis of squaraine dyes, a relevant class of molecules for dye-sensitized solar cell devices, is presented here. Exploiting density functional theory (DFT) in conjunction with second-order vibrational perturbation theory (VPT2), fundamental, overtone, and combination vibrational bands are computed and analyzed, comparing them directly to experimental infrared and Raman spectra. Our results unequivocally demonstrate that VPT2 calculations are mandatory for accurately interpreting the experiments, particularly in the 1100–1650 cm^–1^ region, where anharmonic effects such as frequency shifts, intensity redistribution, and mode couplings are most prominent. Only going beyond harmonic treatment, we were able to undoubtedly identify peculiar vibrational features among symmetric *N*,*N*-disubstituted squaraines and highlight the critical role of low-frequency modes and intramolecular hydrogen-bonding dynamics. These findings provide a refined framework for interpreting coherent vibrational phenomena in squaraine-based molecular systems, offering a transferable computational approach for the spectroscopic characterization of functional chromophores in energy and photonic applications.

## Graphical abstract

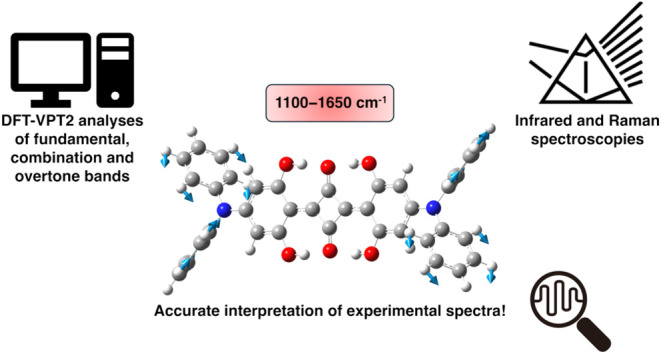

## 1 Introduction

Squaraine dyes constitute a distinctive class of highly conjugated organic chromophores that exhibit intense, narrow absorption features extending well into the near-infrared region.^1–4^ Their characteristic donor–acceptor–donor (D–A–D) architecture promotes extensive intramolecular charge transfer from electron-rich aromatic substituents to the electron-deficient squaric core, yielding pronounced electronic delocalization and a strong sensitivity to environmental perturbations such as solvent polarity, aggregation state, and chemical functionalization.^2–8^ These features have positioned squaraines as promising materials for a broad range of photonic and optoelectronic applications, including organic photovoltaics, bioimaging, and nonlinear optics.^5–11^ In addition to their electronic properties, the vibrational structure of squaraine dyes plays a pivotal role in modulating their optical and spectroscopic response.^11,12^ In both mid-infrared and Raman spectra, these molecules exhibit a dense manifold of vibrational modes that are delocalized across the squaric core, phenolic rings, and exocyclic substituents.^12^ Spectroscopically, this results in highly congested band structures with extensive mode mixing, strong couplings, and the emergence of overtone and combination features. In particular, the Raman-active modes in the 1100–1600 cm^–1^ region frequently exhibit overlapping band patterns involving C=C stretching, C–O bending, and torsional motions of the amino groups.^12^ These interactions can produce intensity borrowing, Fermi resonances, and temperature-dependent shifts that complicate straightforward experimental assignments. In the low-frequency region (below ∼500 cm^–1^), soft torsional and out-of-plane modes involving the exocyclic substituents may induce deviations from planarity and transient symmetry breaking, with potential implications for the photophysical behavior of the dyes in aggregated or confined environments.^11,13,14^

These complexities reveal the limitations of the harmonic approximation that, while computationally affordable, fails to capture the essential anharmonic characteristics of the vibrational manifold in squaraine dyes. Anharmonic effects are especially pronounced in systems that feature flexible moieties, low-frequency deformations, and non-negligible hydrogen bonding, all of which are present in squaraines. As a result, harmonic calculations systematically do not take into account combination bands and overtone intensities and so inaccurately reproduce spectral congestion. Second-order vibrational perturbation theory (VPT2),^15–21^ particularly when based on density functional theory (DFT)-derived quartic force fields, has become a practical and increasingly routine approach for simulating anharmonic vibrational spectra. Its formalism allows the perturbative inclusion of cubic and quartic force constants, capturing frequency shifts, resonance interactions, and spectral intensity redistribution with reasonable computational demands. Alternative methods to perturbative approaches are also possible, such as vibrational self-consistent field,^22^ vibrational configuration interaction,^23^ vibrational coupled clusters,^24^ and dynamics based approaches.^25–30^ These methods, with respect to perturbative approaches, remain more difficult to implement, can be more computationally expensive, and are not easy to use by non-experts, despite recent developments.^31^ Additionally, for a more extensive overview of computational molecular spectroscopy methods, we refer the readers to ref 32. On the other hand, VPT2 achieves a favorable compromise between accuracy and efficiency, making it especially attractive for extended π-conjugated systems and organic dyes where fully variational treatments remain prohibitively expensive. Recent methodological advancements, including automatic resonance detection and robust treatment of near-degenerate states (as in generalized VPT2 schemes, GVPT2),^19,21,33^ have further expanded its applicability to structurally flexible and electronically complex systems. Thus, in this work, we rely on VPT2 calculations that can accurately interpret the experimental IR and Raman vibrational features on a molecular level, particularly in the 1100–1650 cm^–1^region, where anharmonic effects such as frequency shifts, intensity redistribution, and mode couplings are most prominent. In this study, we present a comprehensive anharmonic vibrational analysis of three representative squaraine derivatives (Figure 1): 2,4-bis­[4-(*N*,*N*-diisobutylamino)-2,6- dihydroxyphenyl]­squaraine (**SQ**), 2,4-bis­[4-(*N*,*N*-diphenylamino)-2,6-dihydroxyphenyl]­squaraine (**DPSQ**), and 2,4-bis­[4-(*N*,*N*-dibenzylamino)-2,6-dihydroxyphenyl]­squaraine (**DBSQ**). These molecules serve as prototypical models for a broader class of squaraine-based dyes employed in dye-sensitized solar cells (DSSCs) and related optoelectronic devices. Their structural diversity, ranging from aliphatic to aromatic and benzylic substitution, allows for a systematic exploration of how steric and electronic effects modulate vibrational couplings and anharmonic interactions. Using DFT in conjunction with VPT2, we compute the anharmonic infrared and Raman spectra of all three systems with excellent agreement with the experiments, achieving refined mode assignments. Since anharmonic treatment is still very computationally expensive for the systems analyzed here in this work, we also exploited a computational protocol to define the so-called *active* modes, for which the higher-order differentiation was only carried out. This approach allowed the VPT2-based analysis for the squaraine systems that are quite large, with no further approximation by neglecting the explicit effects of their side chains on the vibrational couplings. On the other hand, knowing combinations and couplings among modes is very crucial, since strong anharmonic couplings between high- and low-frequency vibrational modes can significantly modulate^29,34–37^ or accelerate the nonradiative decay^38^ of photoinduced relaxation in these systems.

**Figure 1:**
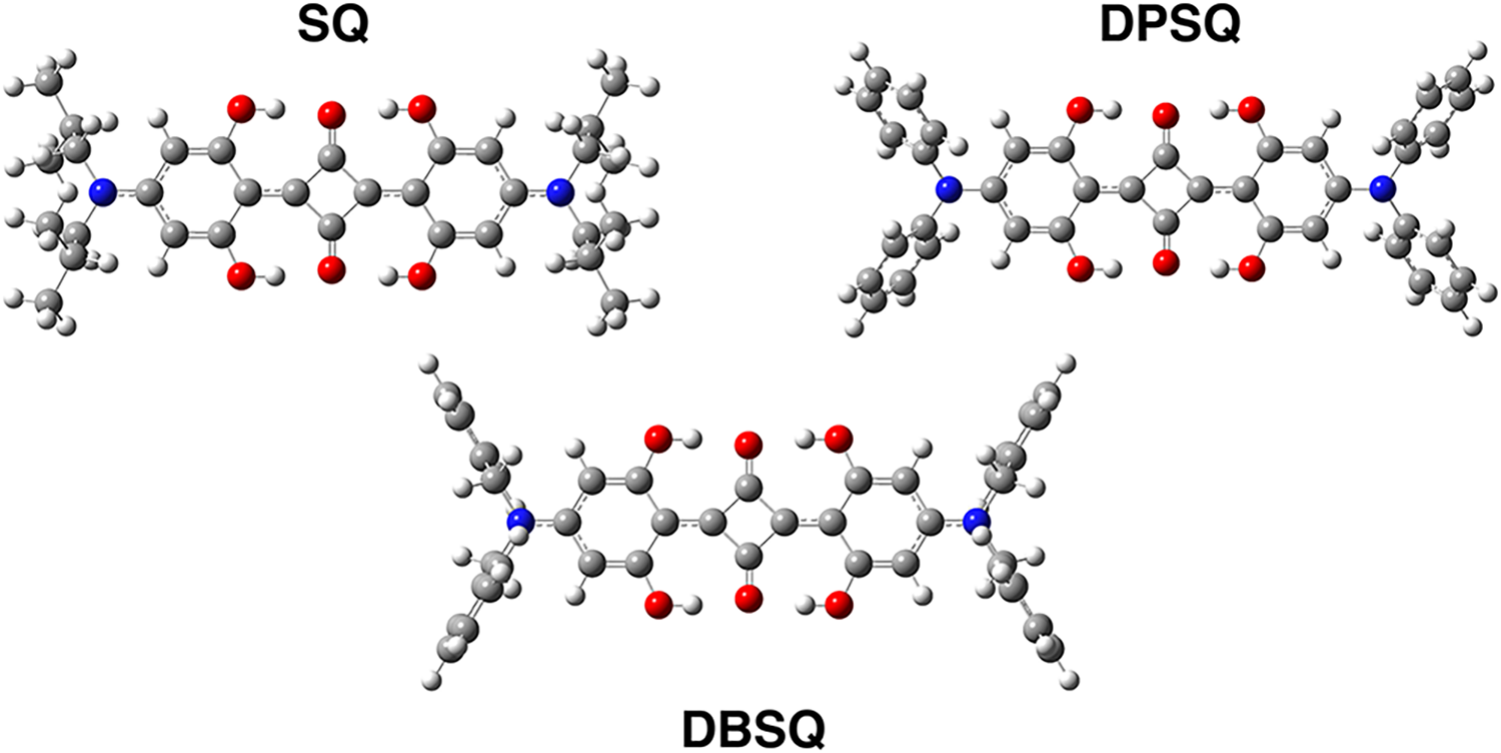
B3LYP/6–31+G­(d,p)/C–PCM minimum energy structures in acetonitrile solution of **SQ**, **DPSQ**, and **DBSQ** dyes. Atoms color palette: C – dark gray; H – white; N – blue; and O – red.

In this work, a direct analysis of fundamental (Fund), overtone (Over), and combination (Comb) vibrational bands on a molecular level for these systems is provided, the critical role of perturbative anharmonic treatments in reproducing and interpreting the vibrational complexity of squaraine dyes is demonstrated, and a transferable computational framework for the spectroscopic characterization of functional chromophores in energy and photonic applications is presented and exploited.

## 2 Materials and Methods

### 2.1 Anharmonic Vibrational Analysis for Large Molecular Systems

Anharmonic vibrational (infrared and Raman) analysis for the three squaraines has been performed and collected by employing the second-order vibrational perturbation theory.^15,17–21^ VPT2 has a good reliability in treating medium-to-large systems and computing vibrational fundamental, combination, and overtone bands.^20,33^ We report here a brief summary of the theory, but we refer the interested readers to more exhaustive and detailed publications.^21,32,33,39^ The vibrational energy terms for a system with *N* internal degrees of freedom, considering only energy minima, are defined^17,33,40,41^

1 $$\mathrm{fundamental}\;\mathrm{bands}:\nu_{1_{i}}=\omega_{i}+2\chi_{\mathrm{ii}}+\frac{1}{2}\sum_{\begin{array}{l} j=1\\ j\neq i \end{array}}^{N}\chi_{\mathrm{ij}}$$

2 $$o\mathrm{vertones}:\nu_{2_{i}}=2\omega_{i}+6\chi_{\mathrm{ii}}+\sum_{\begin{array}{l} j=1\\ j\neq i \end{array}}^{N}\chi_{\mathrm{ij}}$$

3 $$c\mathrm{ombinations}:\;\nu_{1_{i}1_{j}}=\omega_{i}+\omega_{j}+2\chi_{\mathrm{ii}}+2\chi_{\mathrm{ij}}+2\chi_{\mathrm{jj}}+\frac{1}{2}\sum_{\begin{array}{l} k=1\\ k\neq i,j \end{array}}^{N}\left[\chi_{\mathrm{ik}}+\chi_{\mathrm{jk}}\right]=\nu_{1_{i}}+\nu_{1_{j}}+\chi_{\mathrm{ij}}$$

where the anharmonic matrix (**χ**) is defined and can be found in ref 33 and ω_*i*_ is the *i*-th harmonic mode (expressed as cm^–1^). We wish to recall here that the anharmonic matrix elements require that the third and fourth derivatives of the potential energy with respect to the dimensionless normal coordinates ***q*** (i.e., cubic and quartic force constants) are computed. Analogous expressions for the computations of the IR and Raman intensities within this theory have been derived, but we do not report them here, and we refer the readers to the previously mentioned works. We wish to stress here that the computed Raman intensities are considered in the nonresonant regime, since resonant intensities with anharmonic correction are computationally prohibitive. Nevertheless, the obtained results can still provide a valuable framework for interpreting resonant Raman experiments. In VPT2, it is clear that the computational bottleneck is represented by the computation of anharmonic force constants, whose expressions are sometimes numerically evaluated by computing the harmonic force constants at displaced geometries. A method has recently been implemented to reduce computational cost by selecting a subset of normal modes,^39^ performing numerical differentiation only on this selected subset. After computing the harmonic normal modes, one can define the so-called *active* modes; thus, the differentiation is carried out only along these modes, and the anharmonic correction is included in the transition energies of only their fundamental states and overtones. Otherwise, a mode is defined as *inactive* when the anharmonic force constants are not specifically constructed along these modes (saving computational time since no numerical differentiation is performed along these modes). Of course, the anharmonic correction allowing the computation of combination bands is present only for the modes that are *active* (thus, we will show only the combinations among the selected *active* modes). This reduced-dimensionality scheme has been proven promising,^39^ and it is exploited here, given the large size of the analyzed systems (see computational details for the selected *active* modes).

### 2.2 Computational Details

All molecular systems were treated at the DFT level of theory. Electronic structure calculations were accomplished by solving the Kohn–Sham equations using the global hybrid Becke 3-parameter Lee–Yang–Parr, B3LYP,^42–44^ in combination with the 6–31+G­(d,p) basis set.^45–56^ This level of theory was already validated for the characterization of vibrational and optical properties in solution of the selected molecular systems.^12^ Acetonitrile solvent effects were considered by employing implicit solvation models, particularly the conductor-like polarizable continuum model (C–PCM).^57–62^ We chose acetonitrile as an implicit solvent because, in the previous study, the resulting vibrational analyses gave a good agreement with experiments, and to have an easier comparison with previous harmonic results, we kept this choice in the current work. As an additional check, we computed and analyzed the harmonic IR and Raman **SQ** spectra also in gas-phase and implicit cyclohexane and dichloromethane (ϵ = 2.02 and ϵ = 8.93, respectively), reporting the results in the ESI, Figures S2 and S3. An inspection of the results suggests that the choice of the implicit solvent does not significantly affect the agreement with the experimental data; however, acetonitrile demonstrates a very good reproduction of the spectral features, particularly in the **B** region. We noticed that different environments affect mostly the band at about 1275 cm^–1^ (mode # 139, which is mostly a ring breathing of the phenolic rings, see Figure S1) and the band around 1550 cm^–1^ (the squaric C=O stretching, mode # 195, see Figure S1). Geometries were considered fully optimized when both the force (maximum and RMS force, 0.000450 and 0.000300 hartree bohr^–1^ thresholds, respectively) and displacement (maximum and RMS displacement, 0.0018 and 0.0012 bohr thresholds, respectively) values for all atoms were below the threshold criteria. Such geometries are available in the Supporting Information of ref 12. All optimized geometries were checked to be true minima by computing harmonic vibrational frequencies and checking that they were all positive. Harmonic vibrational analysis for infrared and Raman spectra was performed by employing the same level of theory with no further scaling and using the commercial Gaussian 16, version C.01 ^63^ suite of programs. Generalized VPT2 (GVPT2) was employed to deal with the anharmonicity effects on the molecular vibrations for each molecular system^15,17–20^ on a subset of selected *active* normal modes, according to the procedure discussed in the previous paragraph.^39^ If not differently specified, we implied the GVPT2 model when anharmonic results are presented. Thus, we decided to employ the anharmonic vibrational analyses on subgroups of vibrational modes to achieve this at a reasonable computational cost. After performing harmonic analysis, modes were selected as *active* for the subsequent VPT2 calculations by choosing the ones that were more IR or Raman intense in the intricate 1100–1650 cm^–1^ region, along with some of the modes in the lower frequency region involving collective motions. This choice has been also partially guided by the analysis of multidimensional spectra of **SQ** squaraine that presented coupled low- and high-frequency mode contributions to the nonradiative decay for this system facilitating a conical-intersection-driven decay.^64^ The selected *active* Raman modes for each squaraine are displayed in Tables S2, S6, and S9, and selected *active* infrared modes for each squaraine are displayed in Tables S1, S5, and S8. 1–2 Fermi resonances were computed in all of the anharmonic vibrational calculations, without computing the Darling–Dennison (2–2, 1–1, and 1–3) resonances, instead. Raman intensities of computed nonresonant Raman spectra have been used for the figures and were obtained by transforming calculated Raman activities into intensities by assuming a Lorentzian broadening and an incident frequency value of 532 nm. Computed intensities were rescaled to have a better comparison with the experiments; see figure captions for details. As an additional check, to gauge the choice of the modes and the overall protocol, for only one system (**SQ**), we also calculated the full anharmonic spectrum, considering all modes as *active*. In this case, given the arising issue in some of the overlapping frequency terms in the full anharmonic spectrum, we used the deperturbed VPT2 (DVPT2),^65^ that consists of simply removing from the perturbative treatment the resonant terms after their identification. A comparison between the full anharmonic DVPT2 IR spectrum and the chosen subset of *active* mode results (“reduced” DVPT2 and“reduced” GVPT2) for the **SQ** system is reported in Table S3 and Figures S10 and S11. We made sure that the double-ζ 6–31+G­(d,p) basis set provides comparable results in terms of frequencies and intensities with respect to a larger triple-zeta 6–311+G­(d,p) basis set (overall mean frequency difference and in the Raman activity values of 1.62 cm^–1^ and 2.62 Å^6^ were found for the harmonic Raman spectrum of **SQ**, respectively; see also Figure S4 in the ESI). The following anharmonic vibrational analysis has been carried on through a developer version^66^ of the Gaussian suite of programs. For the reader’s clarity, we report that the anharmonic vibrational analysis can be carried out also on the commercial version of the Gaussian suite of programs.^63^ Finally, through the GaussView molecular visualization program,^67^ it was possible to plot infrared and Raman spectra.

### 2.3 Experimental Details

The squaraines **SQ**, **DPSQ**, and **DBSQ** were purchased from Merck and used without further purification. Infrared spectra were recorded from powdered samples embedded in KBr pellets. Raman measurements were performed on squaraine powders pressed into KBr pellets, using a home-built micro-Raman system, as described in ref 12. Nonresonant Raman (NRR) and resonant Raman (RR) spectra were obtained by selecting two excitation sources operating at 514.5 nm (Ar^+^ laser) and 632.8 nm (HeNe laser), respectively. Raman spectra were acquired over the range of 80–4000 cm^–1^ with an instrumental resolution of approximately 2 cm^–1^. However, due to spectral overlap with sample luminescence, RR spectra were reported only up to 1800 cm^–1^. To avoid optical damage occurring at room temperature, the samples were cooled to 100 K in a cryostat cell, and the excitation power was maintained between 0.1 and 0.5 mW during all measurements. For direct comparison of NRR and RR spectra, signal intensities were normalized against a nonresonant internal standard, calcium carbonate (CaCO_3_).

## 3 Results and Discussion

Here, we present a detailed molecular-level analysis of the vibrational features of the studied systems. In a previous publication,^12^ some of the authors reported an initial analysis of the IR and NRR spectra, where it was shown that peculiar IR and Raman-active mode regions are highly sensitive to the conformation, the protonation state, and the nature of the *N*,*N*-disubstituents (these last ones can contribute differently through backbone and collective motions). In particular, **DPSQ** exhibits higher acidity and enhanced conjugate–base stabilization (with respect to the other analyzed squaraines) due to the electron-withdrawing character of the diphenyl substituents, influencing the vibrational and electronic spectral features.^12^ However, that study relied solely on harmonic computations and did not provide a comprehensive peak assignment. Here, we provide a more refined and accurate vibrational analysis with the additional inclusion of the anharmonic contributions to selected fundamental bands along with their intensities. Additionally, we also obtained from VPT2 calculations the combination and overtone bands. This analysis can be very computationally expensive, but it is crucial in the interpretation of multidimensional and time-resolved vibrational spectroscopies.^68–80^ Moreover, Raman spectra under resonant conditions were also recorded. From a theoretical perspective, the presented vibrational analysis, including the anharmonic corrections on the mode frequencies, can be used for interpreting both RR and NRR spectra. Of course, although computed intensities can only be directly compared with NRR intensities, measuring and reporting both experimental RR and NRR spectra is crucial for future work since different enhancements of specific bands can provide an easier detection of some transitions. We wish to recall that in Raman and IR spectra, anharmonicity can usually lead to a red shift of frequencies of overtone and combination bands and may result in intensity redistribution.^81,82^

Experimental IR spectra of the selected prototypical squaraine dyes (Figure 2) can be subdivided into three different spectral regions. Low-frequency collective modes of the *N*,*N*-disubstituents are found in the infrared spectral region (Figure 2), with a frequency range of 350–1100 cm^–1^, namely, region **a**. The second spectral region at 1100–1650 cm^–1^ (namely region **b**) is characterized by the symmetric and asymmetric C–C, C–O, and C–N collective/backbone modes that majorly involve the squaric and phenolic fragments of the molecules.^12^ High-frequency overtones, combination, and O–H and C–H stretching modes are found above 1650 cm^–1^ up to 4000 cm^–1^ and so in the spectral region **c** (Figure 2). In such a spectral region, we focus our anharmonic analysis in the region below 3300 cm^–1^ for the selected squaraine dyes. The anharmonic IR analysis has been centered on the 1100–1650 cm^–1^ frequency range (namely **b**, see Figures 2 and S9) since this spectral region is crucial for also conformational and environmental effects for the selected dyes.^12^ Moreover, thanks to the higher definition of the peaks in such a spectral region, it has been possible to conduct a more detailed vibrational mode analysis. Experimental RR and NRR spectra are displayed in Figure 3 in the range 80–1800 cm^–1^. NRR spectra until 3500 cm^–1^ are reported in Figure S5 (please refer to this last one for the subsequent labeling scheme). The low-frequency region (namely, region **A**, 0–1100 cm^–1^) is more dense with active modes when compared with the IR spectra (Figure 2). In the 1100–1650 cm^–1^ frequency region (namely, region **B**), several symmetric C–C and C–H motions involving the *N*,*N*-disubstituent can be found, already observed for the selected molecules by some of the authors.^12^ Regions **A** and **B** in the spectrum are associated with the collective/backbone modes of the *N*,*N*-disubstituents combined with the hydrogen bond network involving the central fragment of the molecule. The **A** spectral region presents large amplitude motions, and a detailed analysis, both including all modes as *active* and checking the presence of hindered rotors, will be useful in future studies, given also the large changes observed at anharmonic levels for the few *active* modes here studied. On the other hand, in the current work, including such modes is already very useful for understanding their molecular nature along with their effects on **B** regions. At higher energies (from 1650 up to 3300 cm^–1^, namely, region **C**), the spectrum features symmetrical O–H stretching modes localized within the intermolecular hydrogen bond domain (see Figure 1). Anharmonic Raman analysis has been focused on the aforementioned frequency ranges: 0–1100 cm^–1^ (see Figure S6), 1100–1650 cm^–1^ (see Figure S7), and 1650–3300 cm^–1^ (see Figure S8).

**Figure 2:**
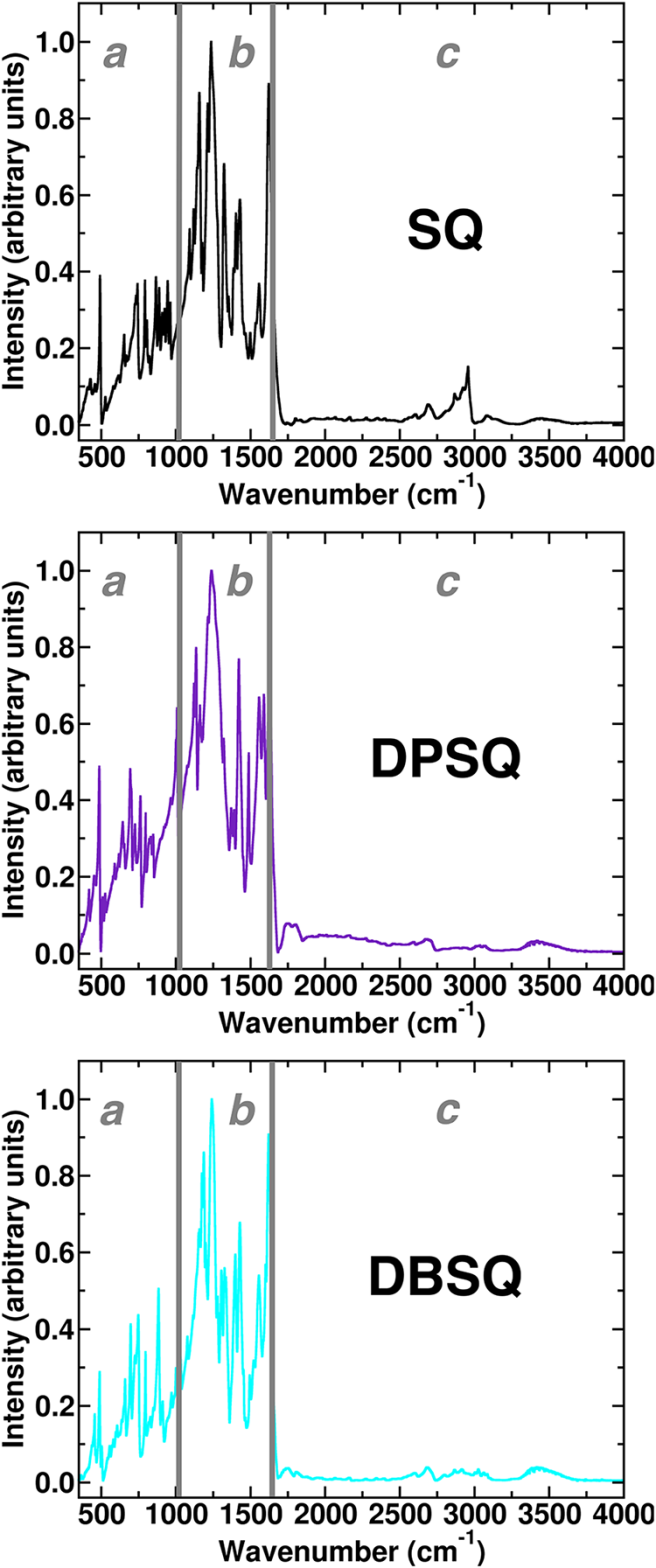
Solid-state experimental IR spectra of **SQ**, black solid line, **DPSQ**, violet solid line, and **DBSQ**, cyan solid line, in the 350–4000 cm^–1^ wavenumber region. Spectral regions of interest are labeled (see the text discussion).

**Figure 3:**
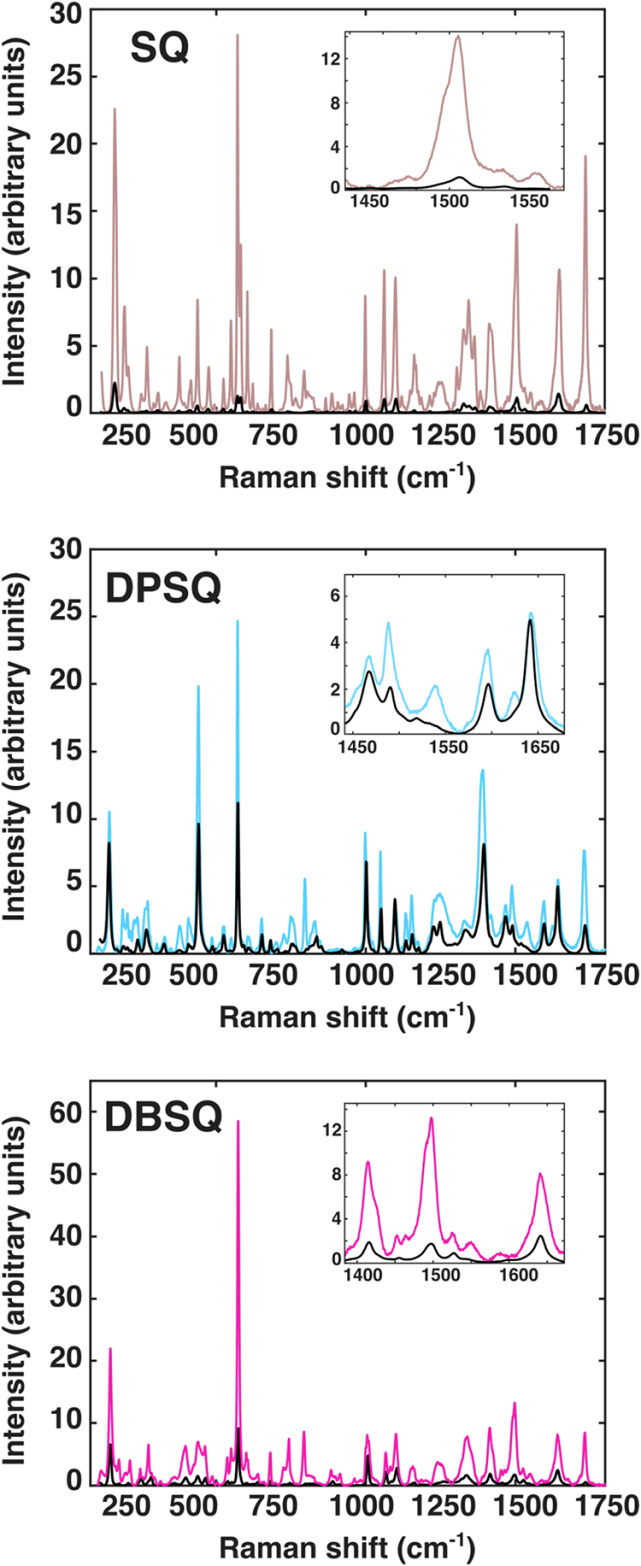
Solid-state experimental resonant Raman spectra of **SQ** (brown solid line), **DPSQ** (light-blue solid line), and **DBSQ** (magenta solid line). In each panel, the corresponding nonresonant Raman spectrum is also shown for comparison (black lines). Spectra are shown over the 0–1800 cm^–1^ wavenumber range. Each panel includes an inset highlighting the combination bands discussed in the main text.

In the following sections, the anharmonic vibrational analyses for **SQ**, **DPSQ**, and **DBSQ** on the selected IR and Raman spectral regions are discussed in detail.

### 3.1 Anharmonic Vibrational Analysis of SQ

After obtaining the harmonic IR spectrum, the anharmonic analysis on the 1100–1650 cm^–1^ (region **B**) wavenumber region for **SQ** has been accomplished by selecting several fundamental modes (see Materials and Methods section and Table S1 for further details). Within this spectral region, the most intense anharmonic mode appears at 1248.91 cm^–1^ (Fund # 139), corresponding to a phenolic ring breathing motion that closely aligns with experimental IR features (Figure S9, black dashed line). Another prominent mode at 1312.57 cm^–1^ (Fund # 147) involves collective O–H and C–H bending spanning nearly the entire molecule and corresponds well with a strong experimental peak at 1322 cm^–1^ (Figure S9, black dashed line). In Table S2, the analyzed harmonic and anharmonic frequencies of the fundamental Raman modes are reported. We begin by focusing on three spectral subregions that exhibit substantial and recurring intensity enhancement moving from NRR to RR spectra: the spectral regions centered at approximately 150, 600, and 1300 cm^–1^. In addition, we also examined the region centered around 1500 cm^–1^, where the combination bands of the 1300 and 150 cm^–1^ modes are expected. Table 1 presents a comparison between the theoretical fundamental modes and the experimental results for **SQ**, highlighting the best matches. In contrast, Table 2 and Table 3 display both combination and fundamental modes that can match the experimentally observed combination modes. As an additional check on the accuracy of the proposed computational protocol, we computed and compared the GVPT2 and the DVPT2 IR spectra for **SQ** for the *active* modes (see Figure S10 and Table S3). Then, we ensure that there is a reasonable agreement of the proposed protocol using a “reduced” number of *active* modes, by computing the DVPT2 anharmonic IR spectra including all of the modes for the **SQ** system (see Figure S11 and Table S3 in the ESI).

**Table 1:** B3LYP/6–31+G­(d,p)/C–PCM Acetonitrile Harmonic and Selected *Active* GVPT2 Anharmonic Nonresonant Raman Fundamental Modes Compared with Experimental Findings for **SQ** ^a^ [Fig fig4] [Fig fig5]

Mode	Proposed mode assignment	Harmonic [cm^–1^, (Å^6^)]	Anharmonic [cm^–1^, (Å^6^)]	Experimental (cm^–1^)
19	β *N*,*N*-disubstituents	152.12 (33.93)	50.30 (74.16)	160
20	β,τ–CH_2_ *N*,*N*-disubstituents	159.04 (3.34)	156.80 (3.68)	
56	β–CH_3_ *N*,*N*-disubstituents	434.49 (11.70)	439.35 (12.38)	436
66	τ phenolic rings	579.79 (25.89)	578.69 (23.27)	571
148	symm δ O–H iHB	1360.84 (26.39)	1315.86 (37.26)	1345
174	symm δ−CH_3_ *N*,*N*-disubstituents	1484.99 (1.52)	1419.79 (1.02)	1415
194	symm δ−CH_2_ *N*,*N*-disubstituents	1536.96 (38.42)	1511.16 (26.85)	1506
196	symm ρ O–H iHB	1554.51 (4.98)	1512.16 (3.78)	

a In parentheses, computed Raman activity is expressed as Å^6^. Raman mode assignment symbols and abbreviations: *β* – breathing; iHB – intramolecular hydrogen bond moiety; *ρ* – rocking; *ν* – stretching; *δ* – scissoring; and *τ* – twisting. The visualization of the selected modes is reported in Figures 4 and 5.

**Table 2:** B3LYP/6–31+G­(d,p)/C–PCM Harmonic and Selected *Active* GVPT2 Anharmonic Nonresonant Raman Fundamental Modes of Interest Compared with the Experimental Modes for the Studied Squaraine Dyes in Acetonitrile^a^

Molecule	Mode	Harmonic [cm^–1^, (Å^6^)]	Anharmonic [cm^–1^, (Å^6^)]	Experimental (cm^–1^)
**SQ**	194	1536.96 (38.42)	1511.16 (26.85)	1506
	196	1554.51 (4.98)	1512.16 (3.78)	
**DPSQ**	177	1523.94 (13.17)	1491.35 (*)	1540
	179	1526.23 (61.26)	1492.43 (23.46)	
	178	1526.12 (0.01)	1494.87 (599.86)	
**DBSQ**	198	1500.50 (35.00)	1460.54 (28.19)	1491

a In parentheses, computed Raman activity is expressed as Å^6^. If the anharmonic intensity diverges, we employed the intensity of the corresponding harmonic calculation for the resulting plots, and we labeled it with an asterisk.

Figures 4 and 5 allow the visualization of some of the selected fundamental vibrational modes. Within the first Raman spectral region **A**, the harmonic analysis shows that the modes at 152.22 and 159.04 cm^–1^ (i.e., Fund # 19,20) could match the experimental mode at about 150 cm^–1^. Such modes are backbone modes of the both *N*,*N*-diisobutyl substituents (see Figure 4 and Table 1 with mode assignment). The anharmonic contribution on the Fund # 19 and 20 modes shows that the Fund # 20 mode better matches the experimental 160 cm^–1^ Raman-active mode (Table 1), while the Fund # 19 mode is drastically red-shifted (50.30 cm^–1^, Table 1). At higher wavenumbers, the experimentally observed mode at 571 cm^–1^ corresponds to the mode identified by both harmonic and anharmonic analyses at about 580 cm^–1^ (Fund # 66, Figure 4 and Table 1). This mode involves a collective motion encompassing the central hydrogen bond network and the twisting of both phenolic fragments. Moving to region **B**, the most notable feature in the experimental RR spectra is an ensemble of bands centered at about 1500 cm^–1^ (see the inset in the upper panel of Figure 3). In particular, a band centered at 1505 cm^–1^ is found, with clear shoulders at 1473, 1496, and 1513 cm^–1^. The anharmonic calculations suggest that the main band could originate from two higher-frequency fundamental modes located at 1511.16 and 1512.16 cm^–1^ (Fund # 194 and # 196, respectively; see Table 2 and Figure 5). The 1511.16 cm^–1^ mode is primarily associated with the symmetric scissoring motion of the −CH_2_ group bonded to the nitrogen atom, while the 1512.16 cm^–1^ mode involves the rocking motion of the four O–H bonds within the central intermolecular hydrogen-bonding network (see Table 1 and Figure 5). In addition to the fundamental modes, significant contributions from combination modes can be identified: the most intense red-shifted shoulder identified in the experimental spectrum most likely corresponds to Comb #20–150, while the weaker, blue-shifted shoulder better aligns with Comb # 19–186, Table 3 and Figure S7. On average, the anharmonic contribution improves the agreement with the experimental data. Particularly, anharmonic selected modes at around 1300–1400 cm^–1^ give comparable peak intensities and band shapes with respect to the NRR experimental spectrum (see Figure S7, solid and dashed green lines). Above 1650 cm^–1^ (namely, in region **C**), high-frequency overtones and combination bands for **SQ** are found. In particular, the anharmonic contribution (Figure S8: left panel, dashed green line) in such frequency range shows a large presence of overtones and combination bands of the selected modes (i.e., Comb # 132–139 and Over # 139 at 2498.54 cm^–1^, see Table S1) with also non-negligible Raman activities: these modes majorly affect the squaric and phenolic rings.

**Figure 4:**
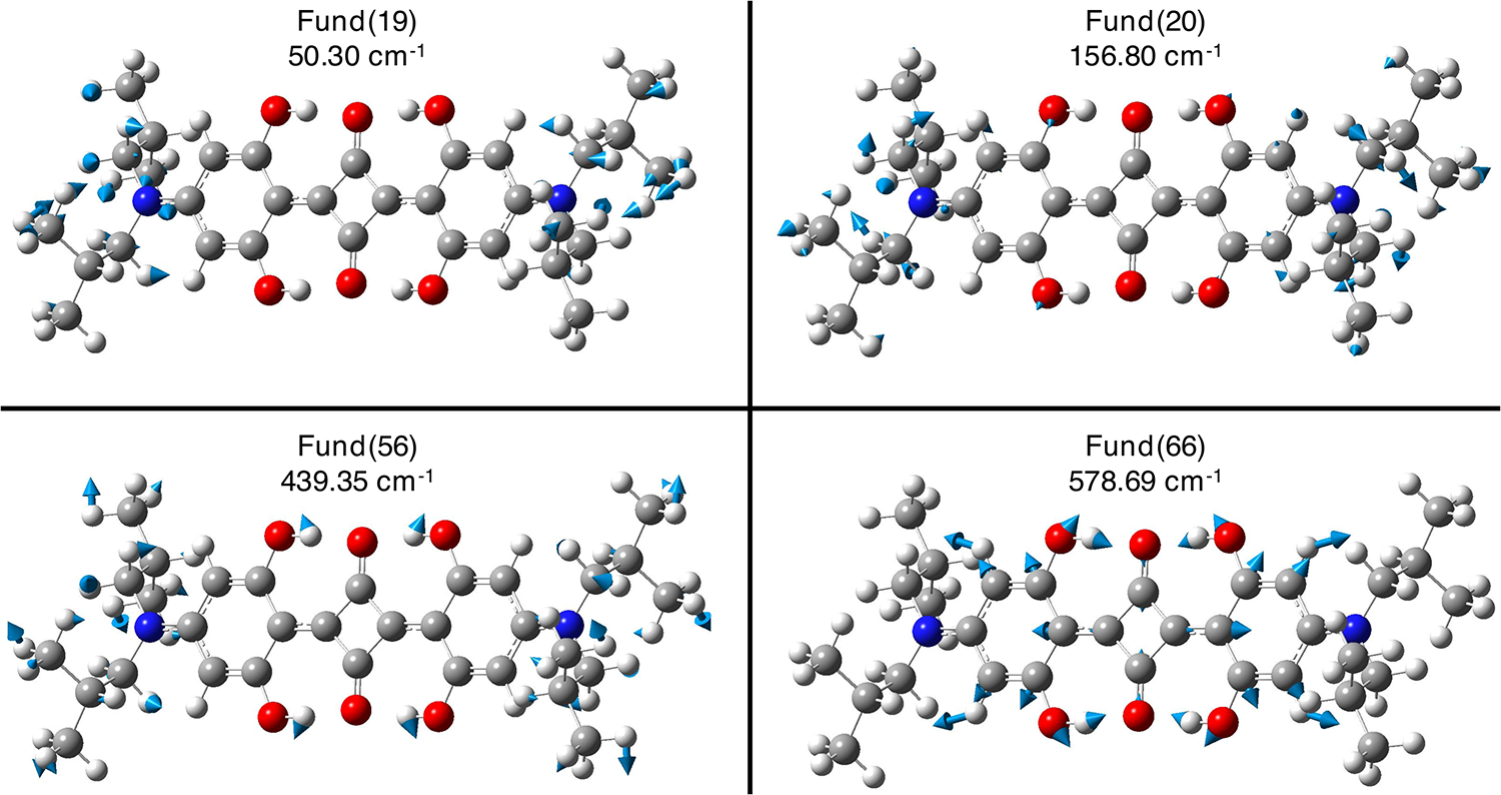
Visualization of B3LYP/6–31+G­(d,p)/C–PCM acetonitrile nonresonant Raman-active low-frequency fundamental modes of **SQ** that better match with experimental observations (please refer to Table 1) with their displacement vectors and anharmonic values. Atoms color palette: C – dark gray; H – white; N – blue; and O – red.

**Figure 5:**
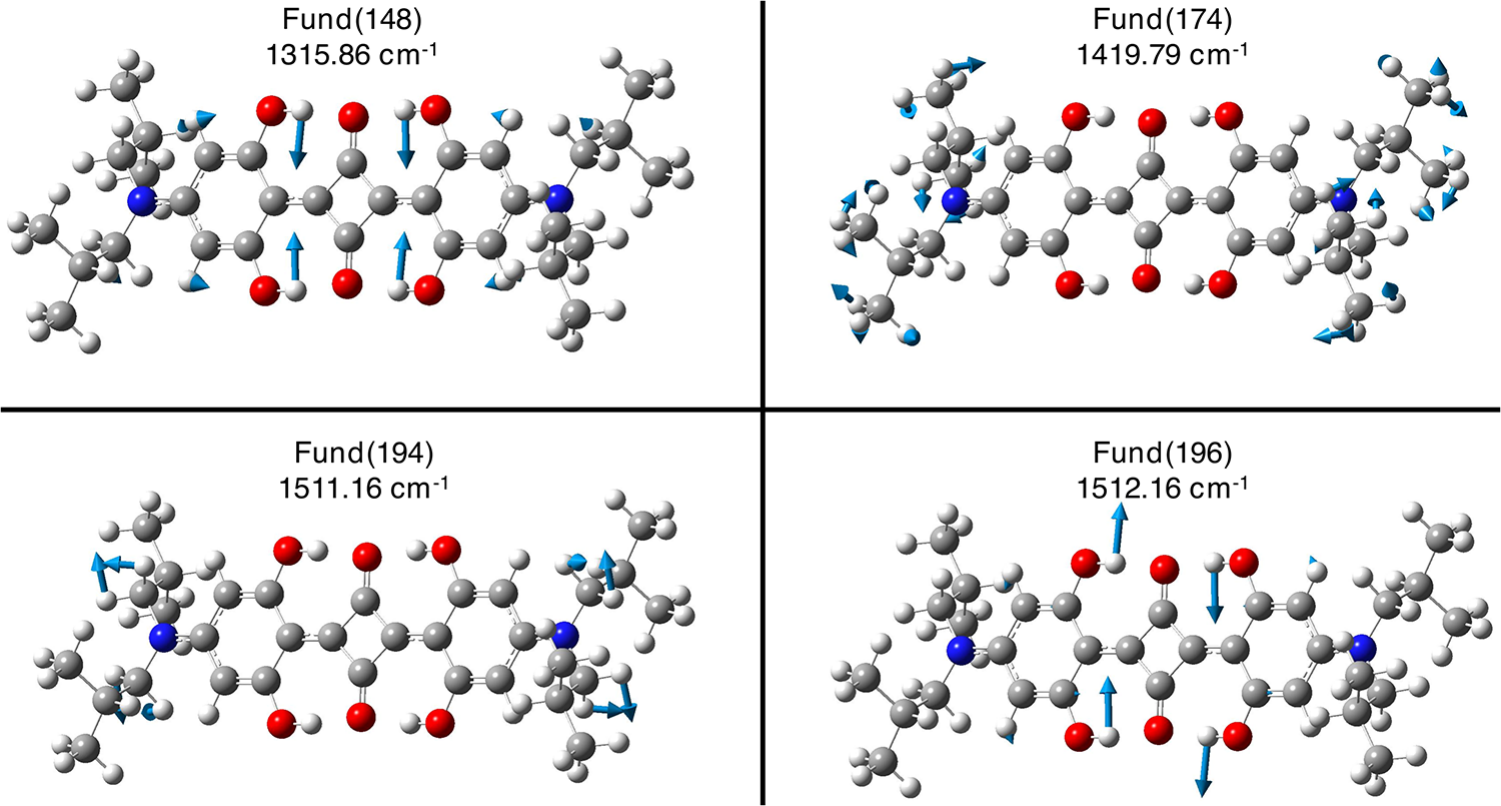
Visualization of B3LYP/6–31+G­(d,p)/C–PCM acetonitrile nonresonant Raman-active higher–frequency fundamental modes of **SQ** that better match with experimental observations (please refer to Table 1) with their displacement vectors and anharmonic values. Atoms color palette: C – dark gray; H – white; N – blue; and O – red.

**Table 3:** B3LYP/6–31+G­(d,p)/C–PCM Anharmonic Nonresonant Raman Combination Modes of Interest Compared with the Experimental Modes for the Studied Squaraine Dyes in Acetonitrile^a^

Molecule	Mode	Anharmonic [cm^–1^, (Å^6^)]	Experimental (cm^–1^)
**SQ**	Comb(20–150)	1491.72 (0.03)	1506
	Comb(19–184)	1499.73 (2.0 · 10^–4^)	
	Comb(19–186)	1516.23 (0.01)	
**DPSQ**	Comb(19–160)	1464.19 (0.12)	1540
	Comb(19–177)	1636.85 (0.02)	
**DBSQ**	Comb(22–198)	1589.61 (2.3 · 10^–3^)	1587

a In parentheses, computed Raman activity is expressed as Å^6^.

### 3.2 Anharmonic Vibrational Analysis of DPSQ

The anharmonic infrared analysis of **DPSQ** (see Table S5 for the selection of the modes considered *active* for the anharmonic treatment and their anharmonic contributions; Figure S9, second panel, blue dashed line) leads to the same observations as the previous molecular system. In particular, the anharmonic spectrum is in good agreement with experiments, and the most intense mode is the one found at 1249.58 cm^–1^ (i.e., Fund # 149, Table S5). Such a vibrational mode is identical to **SQ**, and it seems to be common in this type of squaraine dyes, confirming a conserved vibrational feature among symmetric squaraines.

The selection of Raman modes considered *active* for anharmonic treatment is provided in Table S6. As done for the previous molecular system, in the low-frequency Raman spectral region (Figure S6, middle panel), the experimental ∼150 cm^–1^ mode is found at 142 cm^–1^, which is the collective mode of both *N*,*N*-diphenyl substituents (i.e., Fund # 19, Table S7 and Figure 6). Moreover, it has been found that the experimental mode found at 441 cm^–1^ could be a twisting collective mode of the *N*,*N*-diphenylic substituents with the anharmonic contribution of 408.07 cm^–1^ (Fund # 44, Table S7). Even though we record an underestimation of this mode with respect to the experimental band, which can be due to the theory level, we are confident that the 408 cm^–1^ experimental band can be assigned to the mode Fund # 44, since in this spectral region, similar collective *N*,*N*-disubstituent modes also arise in **SQ**. The ∼590 cm^–1^ experimental mode is found at 572 cm^–1^ (Fund # 57, Table S7 and Figure 6) and corresponds to a collective movement that involves the central hydrogen bond network and the twisting move of both phenolic fragments, as seen for the **SQ** squaraine. Also in this case, the region around 1500 cm^–1^ (region **B**) is interesting. Experimentally (middle panel of Figure 3), a band at 1540 cm^–1^ is emerging in the RR spectrum.

**Figure 6:**
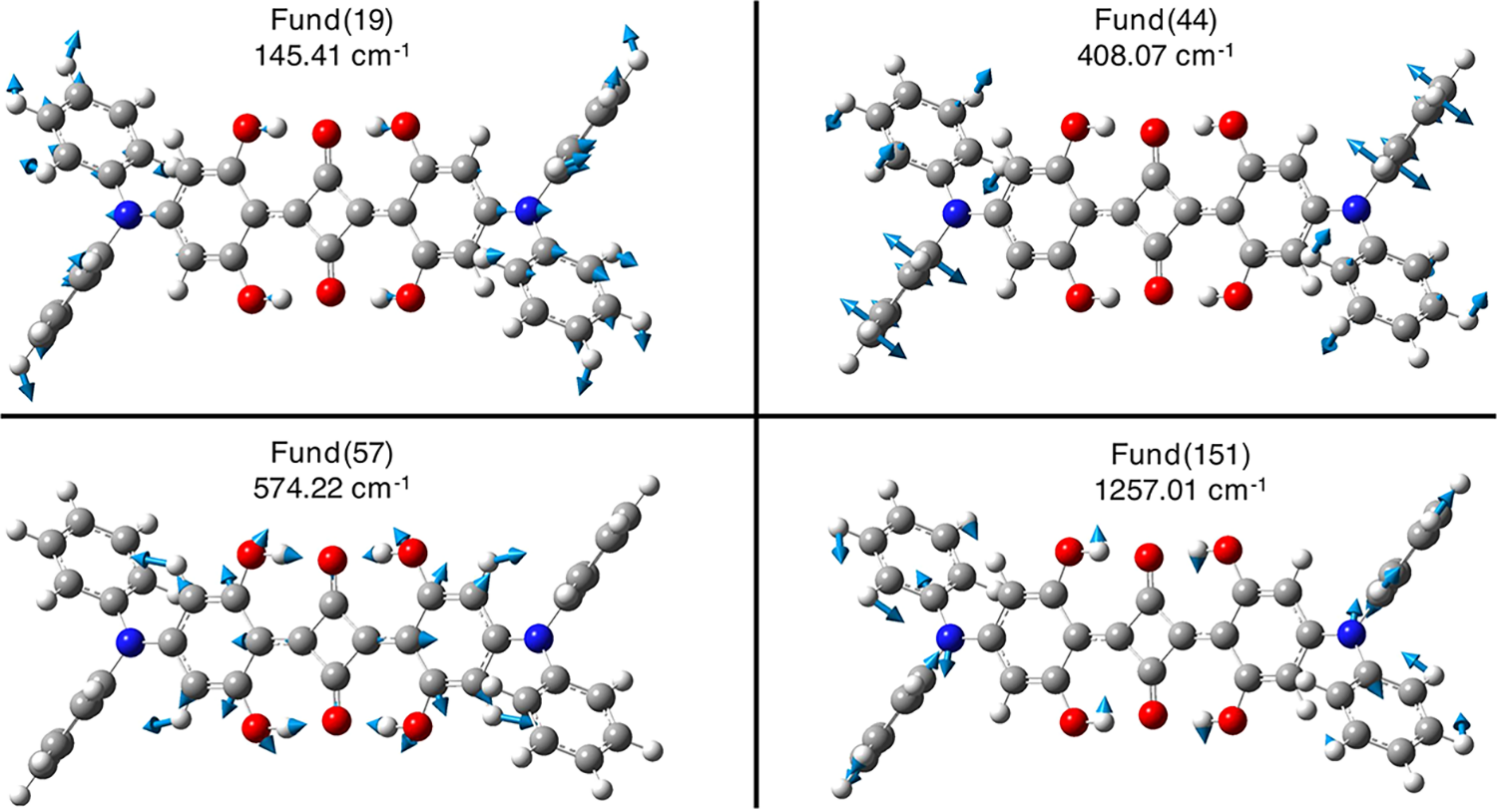
Visualization of B3LYP/6–31+G­(d,p)/C–PCM acetonitrile nonresonant Raman-active low-frequency fundamental modes of **DPSQ** that better match with experimental observations (please refer to Table S7) with their displacement vectors and anharmonic values. Atoms color palette: C – dark gray; H – white; N – blue; and O – red.

Such a mode could match with anharmonic–obtained combination modes that involve the low and higher Raman frequency regions (e.g., Comb # 19–160, see Table 3 and Figures S6 and S7), and also with three higher–frequency modes (Fund # 177,178,179, see Figure 7 for their visualization and Tables 2 and S7). Such modes involve the phenylic −CH scissoring motion in ortho and meta to the nitrogen atom, having different symmetries. The **DPSQ** Raman Fund # 177, 178, and 179 modes show a significantly larger change in Raman activities when the anharmonic treatment is applied, which might be due to the inclusion of some of the resonance effects in the anharmonic treatment. From the vibrational analysis, we found that these modes are involved the most in the experimental band at 1540 cm^–1^, consistently associated with −CH_*n*_ scissoring motions, as previously established for **SQ**. The scissoring O–H bond in the hydrogen bond network for **DPSQ** (see Fund # 160, Table S7 and Figure 7) is found to be mixed with the mode at 145.41 cm^–1^, resulting in the combination mode at 1464.19 cm^–1^ (i.e., Comb # 19–160, see Table 3). This mode was effectively found in the experimental RR spectrum at 1455 cm^–1^. Also, the aforementioned higher-frequency modes that involve the *N*,*N*-diphenylic C–H bonds combine with the low-frequency Fund # 19, resulting in the 1636.85 cm^–1^ combination mode (Comb # 19–177, Table 3). Experimentally, this combination band might correspond to the RR peak observed at 1624 cm^–1^. Above 1650 cm^–1^ and up to 3300 cm^–1^ (Figure S8, dashed red line), high-frequency overtones and combination bands are observed for **DPSQ**. Notably, the anharmonic contribution of **DPSQ** is less intense compared to **SQ**; however, the spectral findings remain consistent with those observed for **SQ**, where the corresponding bands arise from IR-active modes with non-negligible Raman activity (e.g., Over # 149 and Comb # 149–167 found at ∼2500 and 2660 cm^–1^, respectively). The possible minor contribution of anharmonic effects appears to be reflected in the experimental spectra, where the amplitude enhancement observed when moving from RR to NRR for **DPSQ** is the weakest among all of the studied squaraines. A one-to-one comparison between the full harmonic and the selected *active* modes’ anharmonic spectra is not straightforward. Nevertheless, the “reduced” GVPT2 approach is in good agreement with experiments, being capable of providing a molecular interpretation of the vibrational spectrum (improved with respect to the harmonic treatment). As a matter of fact, in the spectral region **B**, we include most of the important modes as *active*, and the analysis shows that the nature of the modes does not change for the included ones. On the other hand, we observed that we are able to reproduce quite well the NRR band shapes in the 1500 to 1600 cm^–1^ region (see Figure S7, middle panel). However, this could also be partially attributed to its electronic transition being the most detuned from the excitation wavelength compared to the other compounds, resulting in a less pronounced amplification of the coupled vibrational modes.

**Figure 7:**
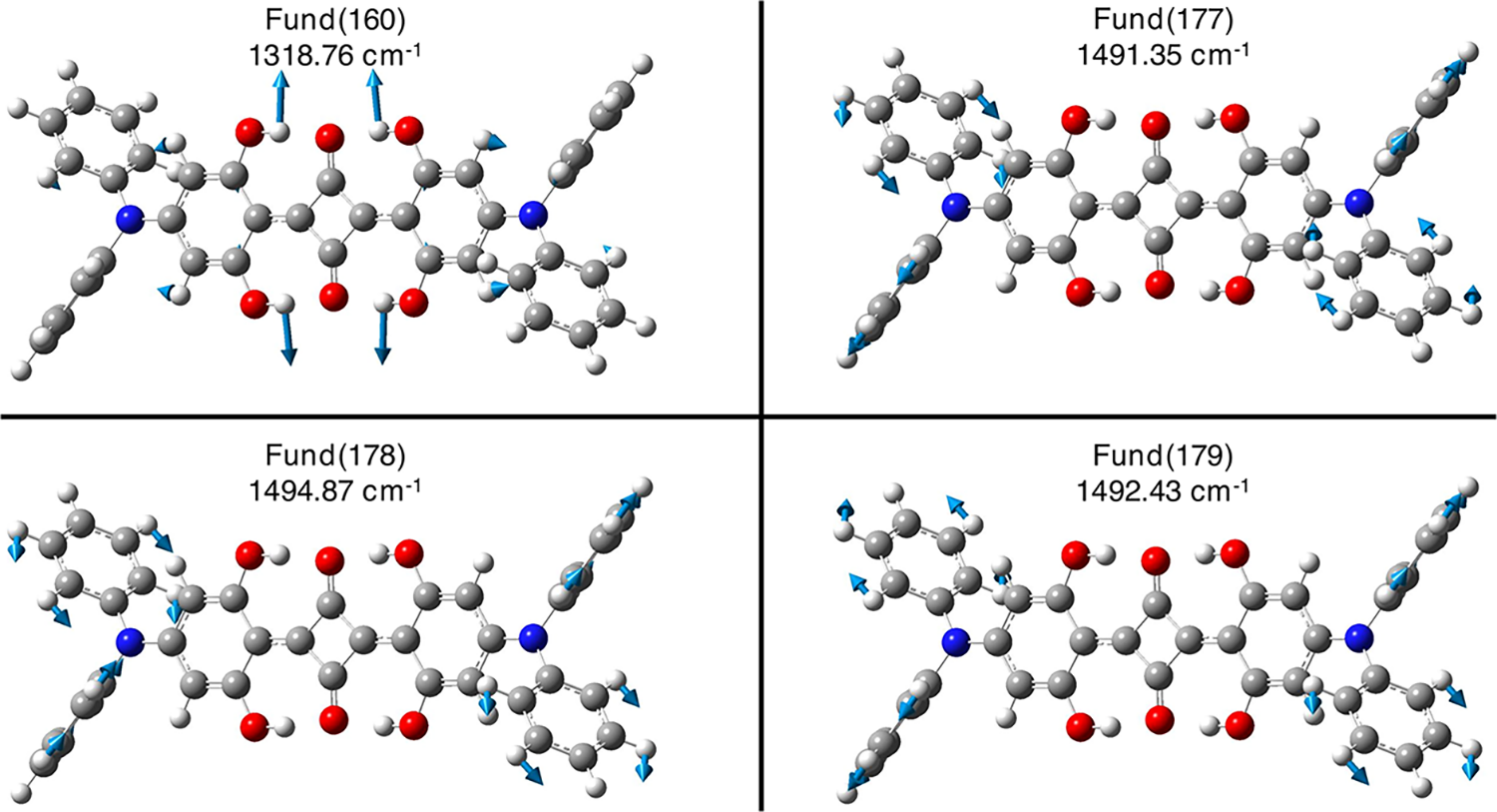
Visualization of B3LYP/6–31+G­(d,p)/C–PCM acetonitrile nonresonant Raman-active higher-frequency fundamental modes of **DPSQ** that better match with experimental observations (please refer to Table S7) with their displacement vectors and anharmonic values. Atoms color palette: C – dark gray; H, white; N, blue; and O – red.

### 3.3 Anharmonic Vibrational Analysis of DBSQ

The **DBSQ** IR modes selection is reported in Table S8. Once the fundamental harmonic modes with potential experimental relevance are selected, the computed anharmonic–contributed spectrum of **DBSQ** (see Figure S9, third panel) exhibits some differences compared to the spectra of the other studied squaraine molecules. The most peculiar intense IR mode (fund no. 167, Figure S9 right panel, dark orange line, and Table S8) with the anharmonic contribution has a very low intensity, but its wavenumber value still matches experiments. The anharmonic IR spectrum suggests that the most intense infrared mode is found at 1181.78 cm^–1^ (Fund # 160); this vibrational mode is the C–H bending of the *N*,*N*-dibenzyl subsituents.^12^ Moreover, it has been found that the mode at 1203.67 cm^–1^ (Fund # 163) is in the same spectral region as Fund # 160; this new mode is the −CH_2_ twisting mode of the *N*,*N*-dibenzyl substituents combined with the C–H bending of the phenolic rings in ortho with respect to the nitrogen atom (for **DBSQ** molecule visualization, see Figure 1).

**DBSQ** anharmonic-computed Raman spectrum shows fewer combination modes since a reduced number of Raman-active fundamental modes were selected. Table S10 presents and compares the theoretical fundamental and combination modes that best match the experimental findings for **DBSQ**, while Table 2 lists the combination and fundamental modes that may correspond to the experimentally observed combination modes. Figure S12 allows the visualization of the selected vibrational modes. On the low-frequency Raman region (i.e., **A**, see Figure S6 magenta dashed line and Figure S5 maroon solid line), the experimental ∼150 cm^–1^ mode is found at 147 cm^–1^, describing the collective mode of aromatic −CH groups for both *N*,*N*-dibenzyl substituents, theoretically found at 129.14 cm^–1^ (see Fund # 22, Table S10 and Figure S12 for its visualization). In the ∼600 cm^–1^ region, the experimental spectrum is dominated by a mode at 575 cm^–1^, which likely corresponds to a theoretical mode calculated at 577.39 cm^–1^ (Fund # 65, see Table S10 and Figure S12). This mode involves a collective motion that includes the central hydrogen bond framework and the twisting of both phenolic fragments, as observed for **SQ** squaraine. It thus represents a characteristic feature of the studied dyes. The experimental RR spectrum of **DBSQ** in the 1500 cm^–1^ region presents two main signals at 1492 and 1498 cm^–1^. These signals match with the fundamental mode calculated at 1460.54 cm^–1^, which is a collective mode of the central hydrogen moiety wagging movement with the – CH_2_ scissoring of the *N*,*N*dibenzyl substituents (see Fund # 198, Figure S12). Such a mode is similar to the higher-frequency 1511.16 cm^–1^ mode of **SQ** (see Fund # 194, Figure 5). In addition, the anharmonic calculation predicts the presence of a combination mode at 1589.61 cm^–1^ (Comb # 22–198), which closely matches an experimental RR weak peak at 1587 cm^–1^. This peak exhibits a relatively low intensity, consistent with the calculated value (see Table 3), and lacks a corresponding feature in the NRR spectrum. Additionally, the collective scissoring mode of X–H bonds (fund # 202, X = C, O), involving the *N*,*N*–dibenzylic substituents and the intramolecular hydrogen bonds, can be assigned to the experimental NRR band found at about 1500 cm^–1^ (see Figures 3 and S7). These results show that the computational protocol can help in the interpretation of the studied Raman spectra.

## 4 Conclusions

In this study, we presented a comprehensive vibrational analysis of three prototypical squaraine dyes: **SQ**, **DPSQ**, and **DBSQ**. We identified conserved vibrational features among the symmetric squaraines with excellent agreement with the experiments. By employing second-order vibrational perturbation theory in conjunction with density functional theory, we have unequivocally demonstrated the necessity and efficacy of an anharmonic treatment for accurately characterizing the complex infrared and Raman spectra of these chromophores. Our results consistently show that VPT2 calculations are crucial to provide reasonable agreement with experimental IR and Raman spectra compared to the harmonic approximation, particularly in the critical 1100 – 1650 cm^–1^ region where anharmonic effects are most pronounced. We identified conserved vibrational features among the symmetric squaraines, such as the intense IR mode around 1249 cm^–1^ (phenolic ring breathing motion) and the collective hydrogen bond network/phenolic twisting motion around 590 cm^–1^ in the Raman spectra. Concurrently, our comparative analysis revealed how subtle structural variations in the *N*,*N*-disubstituents modulate vibrational couplings and anharmonic interactions, providing a detailed understanding of structure–property relationships at the vibrational level. Furthermore, this work unveiled the molecular nature of low-frequency modes (below approximately 500 cm^–1^) and intramolecular hydrogen-bonding dynamics. This detailed understanding is essential for interpreting complex signals in time-resolved spectroscopies and for gaining deeper insights into energy transfer and relaxation pathways in these systems. In summary, this work establishes a robust computational framework for the accurate characterization of functional chromophores, offering valuable contributions to the fields of optoelectronics and spectroscopy.

## Supplementary Material



## Data Availability

The data that support the findings of this study are available within the Supporting Information and from the corresponding author upon reasonable request.

## Notes

Mean frequency and intensity differences evaluation procedures; analysis of the effects of different environments and different basis sets on the vibrational spectra of **SQ**; experimental solid-state nonresonant Raman spectra, experimental and calculated IR and Raman spectra additional data, list of the *active* IR and Raman modes for the anharmonic treatment, **DPSQ** and **DBSQ** Raman modes with their proposed assignments and their normal mode visualization; comparison of the DVPT2 and GVPT2 models on the IR spectrum of **SQ** compared with experimental solid-state IR and calculated harmonic spectra; and mean frequency and intensity differences evaluations of the several anharmonic approaches; Regarding minimum energy structure coordinates, please refer to ref 12 (PDF)
