# Supplementary material for: Spectroscopic Exploration of Squaraine Dyes: Molecular Characterization of Fundamental, Combination, and Overtone Bands
Source: ACS Phys Chem Au. 2025 Dec 18;6(1):69–80. doi: 10.1021/acsphyschemau.5c00079 (PMC12856650; doi:10.1021/acsphyschemau.5c00079)
Supplement: Supplementary file 1 [file pg5c00079_si_001.pdf]

Supporting Information: Spectroscopic Exploration of Squaraine Dyes: Molecular Characterization of Fundamental, Combination, and Overtone Bands

Edoardo Buttarazzi,[†,‡] Vittoria Burigana,[¶] Elisabetta Collini,[¶] and Alessio Petrone[*,§,†,‖]

†*Scuola Superiore Meridionale, Largo San Marcellino 10, I-80138, Napoli, Italy*

‡*Department of Chemical Sciences, University of Napoli Federico II, Complesso Universitario di Monte S. Angelo, via Cintia 21, I-80126, Napoli, Italy*

¶*Department of Chemical Sciences, University of Padova, Via Marzolo 1, I-35131 Padova, Italy*

§*Department of Chemical Sciences, University of Napoli Federico II, Complesso Universitario di Monte S. Angelo, Via Cintia 21, I-80126, Napoli, Italy*

‖*Istituto Nazionale Di Fisica Nucleare, sezione di Napoli, Complesso Universitario di Monte S. Angelo ed. 6, Via Cintia, I-80126, Napoli, Italy*

E-mail: alessio.petrone@unina.it

1 Mean Differences Evaluation

The mean frequency difference was evaluated as the average of the differences between the frequency values in the dataset and those in the chosen reference. The mean intensity difference was evaluated by first normalizing the intensity values of each dataset with respect to its maximum, matching intensities between corresponding modes (analyzing their displacement vectors) in each dataset, retaining only normalized intensities greater than *0.01*, and then transforming the normalized intensities in the relative percentage difference for each i-th retained value (x_i) with respect to the i-th reference (x_i^{ref}), according to the following expression: $[100 \cdot (x_i - x_i^{ref}) / x_i^{ref}]$. Finally, we report the average as the mean intensity difference.

2 Effects of Different Environments on the Computed Spectra

B3LYP/6−31+G(d,p)/C−PCM harmonic IR spectra in different environments and gas-phase are compared with solid–state experiments for the **SQ** system in fig. S2. Taking acetonitrile solvent as reference, we observed a mean frequency difference in the whole spectral region of $\sim +8\,\mathrm{cm}^{-1}$ in gas, of $\sim +1\,\mathrm{cm}^{-1}$ in dichloromethane, and of $\sim +4\,\mathrm{cm}^{-1}$ in cyclohexane. In the **b** spectral region, we observed a mean frequency difference of $\sim +7\,\mathrm{cm}^{-1}$ in gas, $\sim +4\,\mathrm{cm}^{-1}$ in cyclohexane and $\sim +1\,\mathrm{cm}^{-1}$ in dichloromethane. In such a region, the mean intensity difference (on the normalized intensities) is $\sim +28\%$ in gas, $\sim +21\%$ in cyclohexane and $\sim +3\%$ in dichloromethane. In particular, the band at about $1275\,\mathrm{cm}^{-1}$ (mode # 139) which is mostly a ring breathing of the phenolic rings, and the band around $1550\,\mathrm{cm}^{-1}$, the squaric C=O stretching (mode # 195), are the ones mostly affected in both intensities (larger) and frequencies (higher) when computed in gas phase.

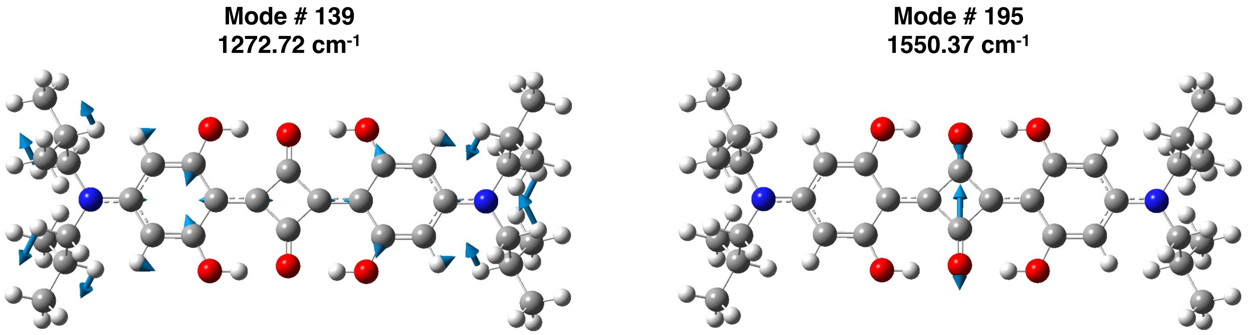

Figure S1: Visualization of B3LYP/6−31+G(d,p)/C−PCM IR modes of **SQ** in acetonitrile that are particularly affected by different environments and gas phase (please refer to Figure S2 for IR spectra). Displacement vectors and harmonic values in acetonitrile solvent are provided. Atoms color palette: C-dark gray; H-white; N-blue; and O-red.

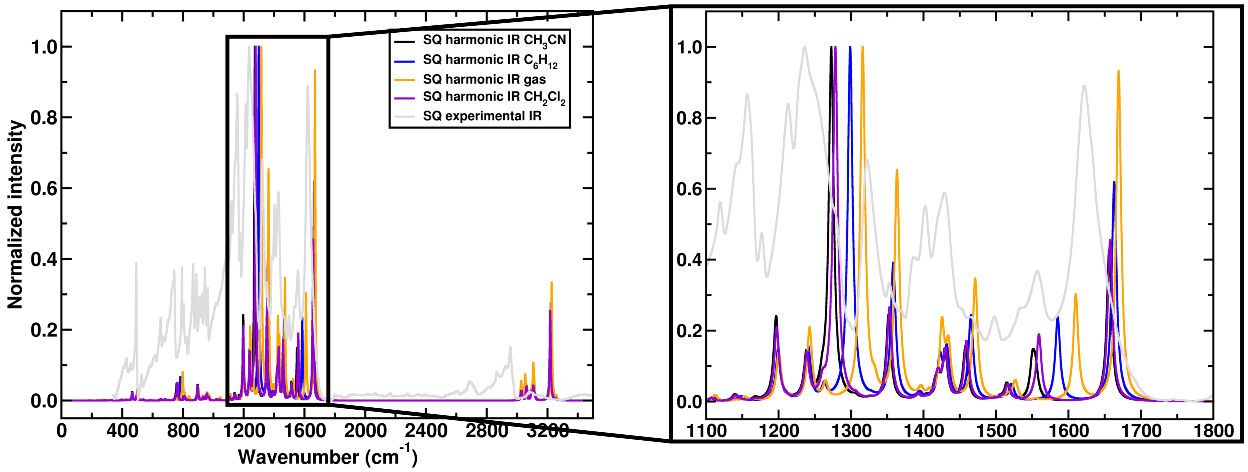

Figure S2: B3LYP/6−31+G(d,p)/C−PCM harmonic IR and gas-phase spectra compared with solid–state experiments (solid gray line) for the **SQ** system. The $1100-1800\,\mathrm{cm}^{-1}$ (namely region **b**) spectral region magnification is also reported. Implicit solvents are used within the C−PCM model: acetonitrile (solid black line, $\epsilon = 35.69$), dichloromethane (solid violet line, $\epsilon = 8.93$), and cyclohexane (solid blue line, $\epsilon = 2.02$); gas–phase (solid orange line) results are also reported. Both experimental and computed intensities were uniformly scaled to obtain the most intense band ($\sim$1300 cm^{-1}) to unity for a better comparison.

B3LYP/6−31+G(d,p)/C−PCM harmonic NRR spectra in different environments and gas-phase are compared with solid–state experiments for the **SQ** system in fig. S3. Taking acetonitrile solvent as reference we observed a mean frequency difference in the entire range of $\sim +8\,\mathrm{cm}^{-1}$ **SQ** in gas, $\sim +1\,\mathrm{cm}^{-1}$ **SQ** in dichloromethane and $\sim +4\,\mathrm{cm}^{-1}$ **SQ** in cyclohexane.

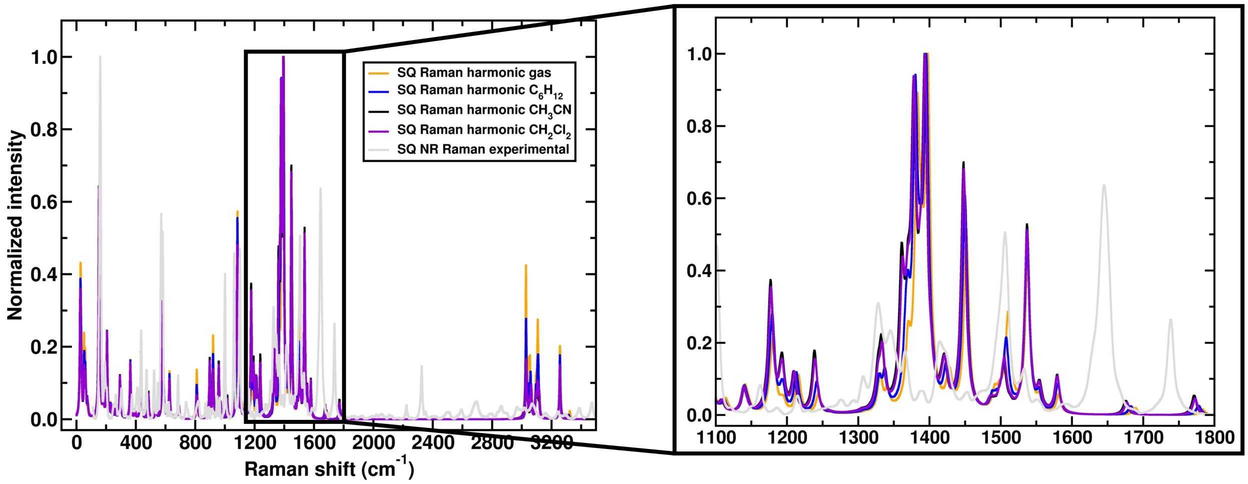

Figure S3: B3LYP/6−31+G(d,p)/C−PCM harmonic NRR spectra and gas-phase compared with solid–state non–Resonant Raman spectrum (solid gray line) for the **SQ** system. The $1100-1800\,\mathrm{cm}^{-1}$ (namely region **B**) spectra region magnification is also reported. Implicit solvents are used within the C−PCM model: acetonitrile (solid black line, $\epsilon = 35.69$), dichloromethane (solid violet line, $\epsilon = 8.93$), and cyclohexane (solid blue line, $\epsilon = 2.02$); gas–phase (solid orange line) results are also reported. Both experimental and computed intensities were uniformly scaled to obtain the most intense band to unity for a better comparison.

3 Effects of Different Basis Sets on the Computed Spectra

B3LYP/6−31+G(d,p)/C−PCM and B3LYP/6−311+G(d,p)/C−PCM harmonic non–Resonant Raman spectra comparison is reported in fig. S4 for **SQ**. The triple–ζ basis set shows a mean frequency difference of $\sim +2\,\mathrm{cm}^{-1}$, in the whole 0 to $3500\,\mathrm{cm}^{-1}$ frequency range, and of $\sim -4\,\mathrm{cm}^{-1}$ in the spectral region **B**. The mean intensity difference in such a region, although larger ($\sim +19\%$) if compared to the frequencies, is still reasonable and is mostly due the discrepancies in the modes ~ 1325 and $\sim 1360\,\mathrm{cm}^{-1}$.

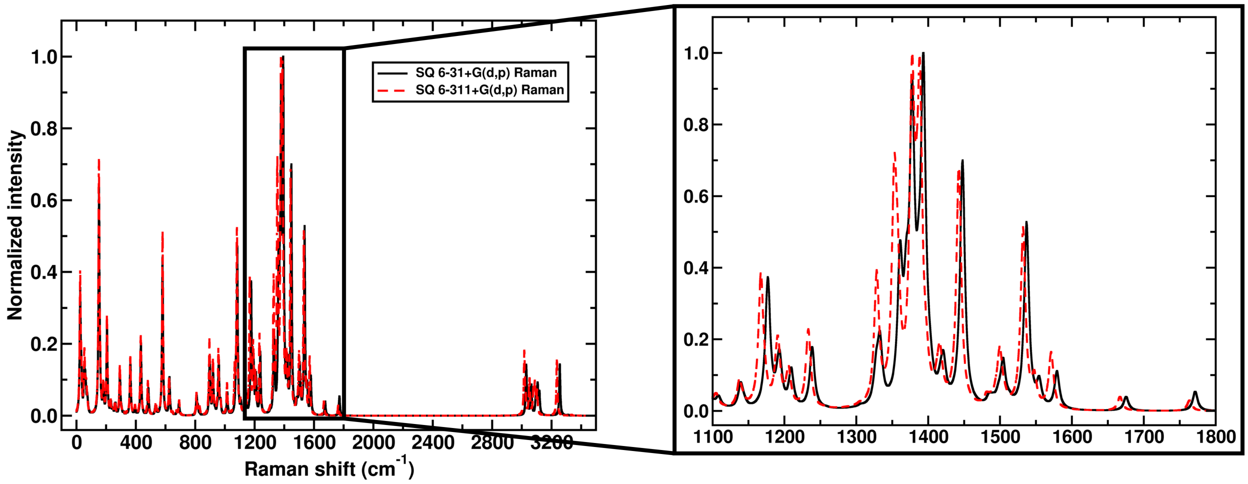

Figure S4: B3LYP/6−31+G(d,p)/C−PCM (solid black line) and B3LYP/6−311+G(d,p)/C−PCM (dashed red line) harmonic non–Resonant Raman spectra for the **SQ** system using acetonitrile as implicit solvent. The $1100 - 1800\,\mathrm{cm}^{-1}$ (namely region **B**) spectra region magnification is also reported. Computed intensities were uniformly scaled to obtain the most intense band to unity for a better comparison.

4 Experimental Solid-State Non–Resonant Raman Spectra

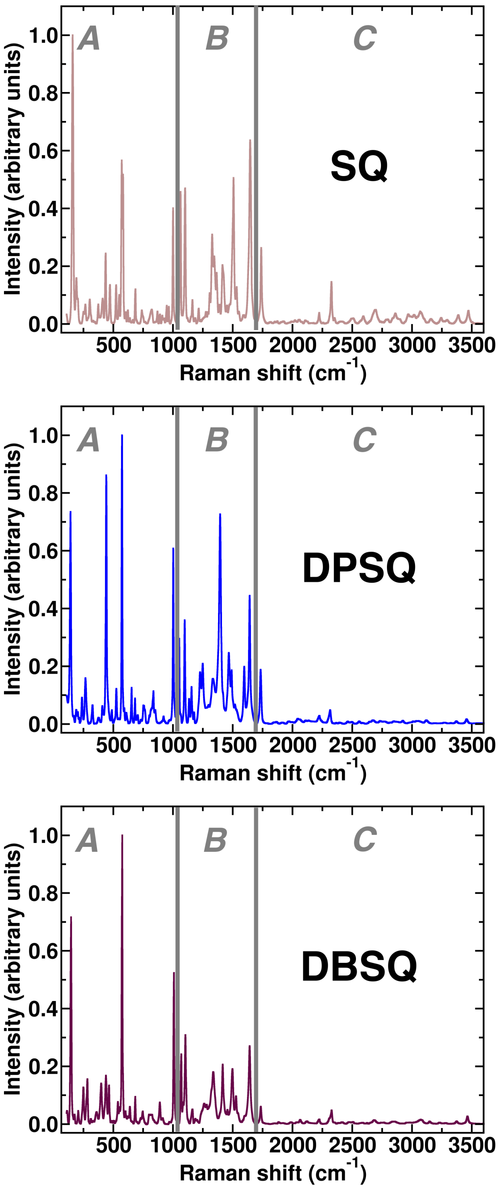

Figure S5: Solid–state experimental non–Resonant Raman spectra of **SQ**, brown solid line, **DPSQ**, blue solid line, and **DBSQ**, maroon solid line, in the $0 - 3600\,\mathrm{cm}^{-1}$ wavenumber region. Spectral regions of interest are labeled (see main text discussion).

5 Experimental and Computed Non–Resonant Raman Spectra on Three Spectral Regions

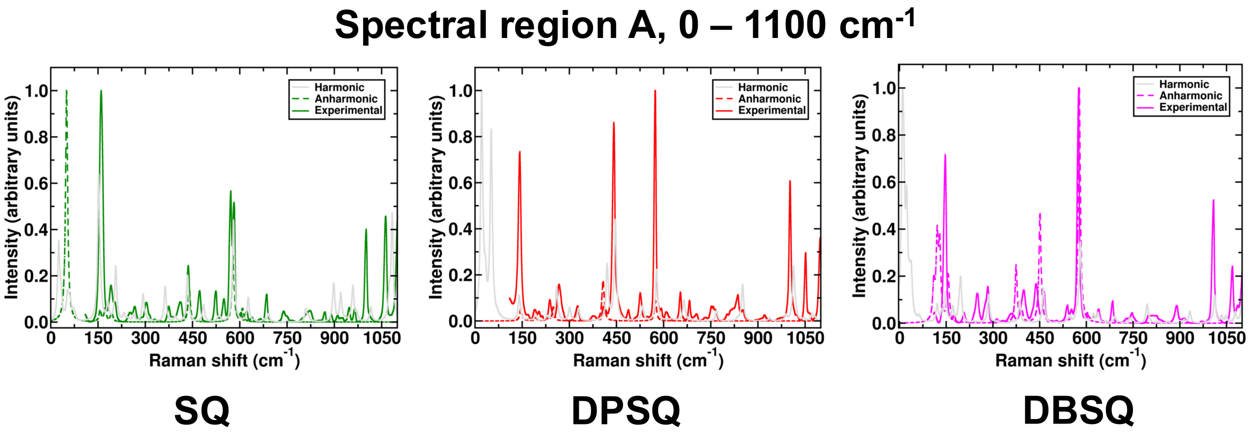

Figure S6: Non–Resonant Raman spectra at $0 - 1100\,\mathrm{cm}^{-1}$ frequency range (namely **A**, Figure S5). Solid state Raman (solid lines) and B3LYP/6−31+G(d,p)/C−PCM acetonitrile harmonic (solid grey line) and anharmonic (dashed lines) Raman spectra of **SQ** (green), **DPSQ** (red) and **DBSQ** (magenta). Please refer to Tables S2, S6 and S9 for mode selection. Computed intensities were uniformly scaled to obtain the most intense band to unity for a better comparison. Harmonic and experimental data are from Ref. 1.

Spectral region B, 1100 – 1650 cm^{-1}

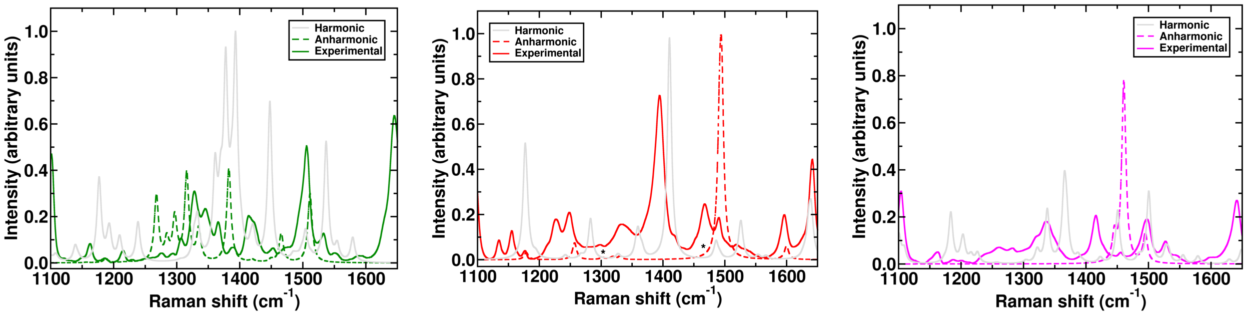

SQ **DPSQ** **DBSQ**

Figure S7: Non–Resonant Raman spectra at $1100 - 1650\,\mathrm{cm}^{-1}$ frequency range (namely **B**, Figure S5). Solid state Raman (solid lines) and B3LYP/6−31+G(d,p)/C−PCM acetonitrile harmonic (solid grey line) and anharmonic (dashed lines) Raman spectra of **SQ** (green), **DPSQ** (red) and **DBSQ** (magenta). Please refer to Tables S2, S6 and S9 for mode selection and for the divergent anharmonic intensities (in these cases, we report the intensity of the corresponding harmonic calculation and we labeled it with an asterisk in the resulting figures). Computed intensities were uniformly scaled to obtain the most intense band to unity for a better comparison. Harmonic and experimental data are from Ref. 1.

Spectral region C, 1650 – 3300 cm^{-1}

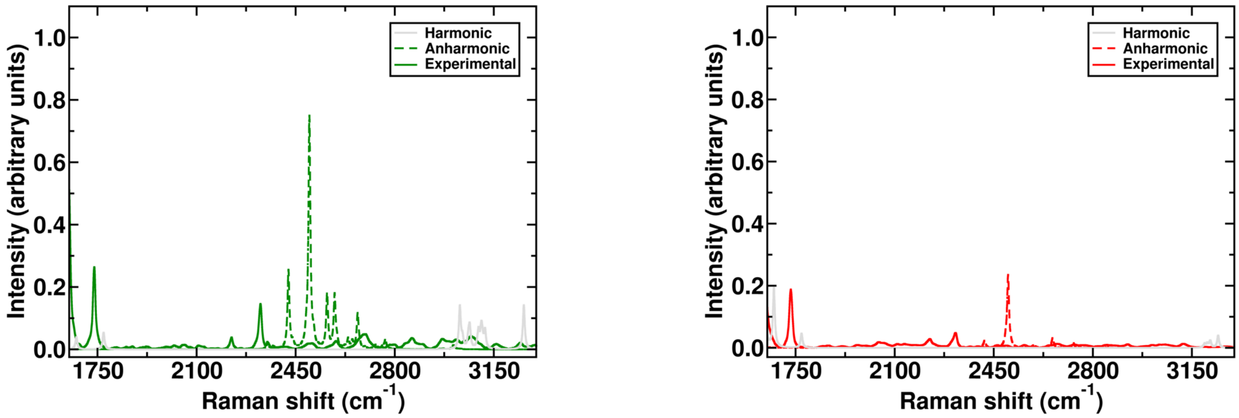

SQ **DPSQ**

Figure S8: Non–Resonant Raman spectra at $1650 - 3300\,\mathrm{cm}^{-1}$ frequency range (namely **C**, Figure S5). Solid state Raman (solid lines) and B3LYP/6−31+G(d,p)/C−PCM acetonitrile harmonic (solid grey line) and anharmonic (dashed lines) Raman spectra of **SQ** (green) and **DPSQ** (red). Please refer to Tables S2 and S6 for mode selection. Due to low–intensity spectrum, anharmonical combination and overtone bands spectral region of **DBSQ** is not displayed. Computed intensities were uniformly scaled to obtain the most intense band to unity for a better comparison. Harmonic and experimental data are from Ref. 1.

6 Experimental and Computed Infrared Spectra on the *b* Spectral Region

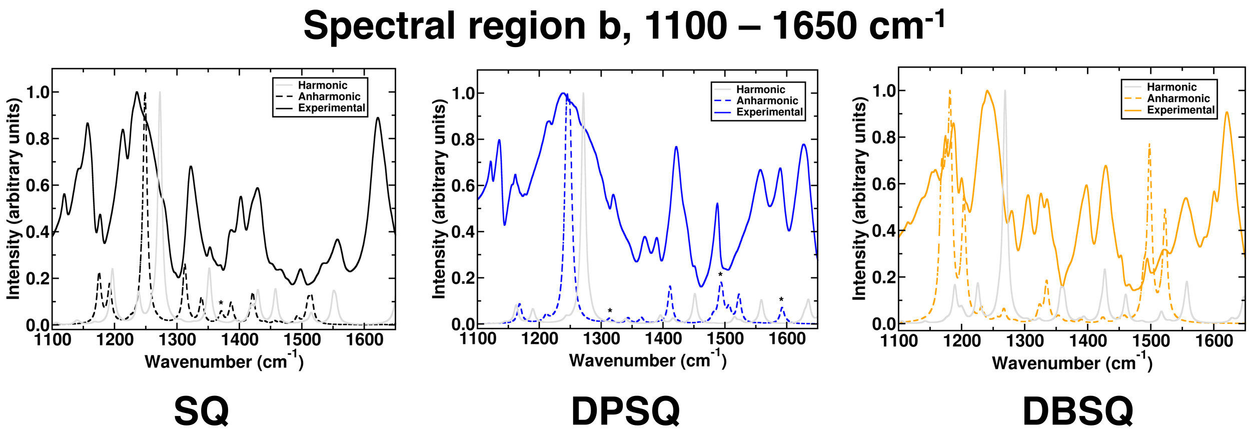

Figure S9: Infrared spectra at $1100 - 1650\,\mathrm{cm}^{-1}$ frequency range. Solid state IR (solid lines) and B3LYP/6−31+G(d,p)/C−PCM acetonitrile harmonic (solid grey line) and anharmonic (dashed lines) infrared spectra of **SQ** (black), **DPSQ** (blue) and **DBSQ** (orange). Please refer to Tables S1, S5 and S8 for mode selection and for the divergent anharmonic intensities (in these cases, we report the intensity of the corresponding harmonic calculation and we labeled it with an asterisk in the resulting figures). Computed intensities were uniformly scaled to obtain the most intense band to unity for a better comparison. Harmonic and experimental data are from Ref. 1.

7 Harmonic and Anharmonic Infrared and Non–Resonant Raman fundamental Modes of SQ

Table S1: B3LYP/6−31+G(d,p)/C−PCM harmonic and selected *active* anharmonic fundamental infrared modes of **SQ** in acetonitrile. In parenthesis, computed infrared intensities are expressed as km/mol. If the anharmonic intensity diverges, we employed the intensity of the corresponding harmonic calculation for the resulting plots and we labeled it with an asterisk in Figure S9, dashed black line.

Mode	Harmonic [cm^{-1}, (km/mol)]	Anharmonic [cm^{-1}, (km/mol)]
132	1196.64 (3469.60)	1175.30 (2669.92)
133	1209.45 (53.90)	1187.18 (58.65)
135	1238.43 (1869.90)	1192.25 (1964.85)
138	1260.18 (556.63)	1242.38 (532.44)
139	1272.72 (14623.65)	1248.91 (12182.49)
141	1305.81 (109.43)	1225.46 (110.42)
143	1326.46 (39.89)	1268.40 (35.28)
147	1351.81 (3527.87)	1312.57 (3125.65)
155	1386.76 (27.04)	1296.82 (9.71)
157	1392.85 (199.87)	1300.02 (41.15)
160	1402.29 (29.36)	1368.25 (∗)
163	1415.77 (420.77)	1371.10 (590.77)
165	1419.90 (722.39)	1386.83 (1092.32)
169	1429.70 (2001.52)	1339.34 (1357.80)
171	1457.80 (2214.77)	1421.40 (1637.81)
185	1498.59 (56.59)	1456.39 (155.19)
189	1506.30 (96.30)	1465.19 (103.53)
192	1515.04 (700.16)	1492.69 (373.73)
195	1550.37 (1568.46)	1510.06 (1003.28)
197	1554.65 (1121.04)	1515.60 (1181.17)
198	1560.16 (30.85)	1519.13 (30.02)

Table S2: B3LYP/6−31+G(d,p)/C−PCM harmonic and selected *active* anharmonic fundamental non–Resonant Raman modes of **SQ** in acetonitrile. In parenthesis, computed Raman activity is expressed as Å^6.

Mode	Harmonic $[cm^{-1}, (\text{Å}^6)]$	Anharmonic $[cm^{-1}, (\text{Å}^6)]$
19	152.12 (33.93)	50.30 (74.16)
20	159.04 (3.34)	156.80 (3.68)
23	181.18 (3.91)	172.69 (4.13)
25	206.27 (13.05)	191.38 (1.46)
28	229.91 (0.02)	175.57 (0.20)
30	232.35 (1.70)	195.16 (1.73)
56	434.49 (11.70)	439.35 (12.38)
57	446.74 (0.09)	435.66 (0.13)
65	579.06 (0.51)	577.87 (0.46)
66	579.79 (25.89)	578.69 (23.27)
71	626.64 (6.25)	609.54 (2.91)
144	1327.70 (5.95)	1284.29 (8.67)
146	1332.96 (11.84)	1267.78 (25.77)
148	1360.84 (26.39)	1315.86 (37.26)
150	1369.53 (16.07)	1334.89 (13.47)
170	1447.77 (49.79)	1382.71 (40.21)
174	1484.99 (1.52)	1419.79 (1.02)
184	1498.27 (1.53)	1449.86 (1.25)
186	1504.25 (8.64)	1465.88 (2.42)
194	1536.96 (38.42)	1511.16 (26.85)
196	1554.51 (4.98)	1512.16 (3.78)

Table S3: B3LYP/6−31+G(d,p)/C−PCM acetonitrile anharmonic IR analysis comparison for the *active* modes of **SQ**. Results obtained using all modes as *active* (full DVPT2, DVPT2f), or the just the selected subset at both DVPT2 (reduced DVPT2, DVPT2r) and GVPT2 (reduced GVPT2, GVPT2r) levels are presented (please refer to Table S1 for the selected subset). Frequencies are expressed as cm^{-1} and intensities as km/mol. If the anharmonic intensity diverges, we labeled it with an asterisk and we used the intensity of the corresponding harmonic calculation in plotting the spectra in Figures S9 to S11. We also reported for completeness in the table the modes labeled with the latin letters since they are the modes that became most intense in the full DVPT2 approach (previously with null IR intensity in the harmonic calculation, thus not considered in the selection of *active* IR modes). Please refer to Table S4 for the mean intensities and frequencies differences between the several anharmonic models.

Mode	$\nu_{IR,harm}$	$\nu_{IR,GVPT2r}$	$\nu_{IR,DVPT2r}$	$\nu_{IR,DVPT2f}$	$I_{IR,harm}$	$I_{IR,GVPT2r}$	$I_{IR,DVPT2r}$	$I_{IR,DVPT2f}$
132	1196.64	1175.30	1175.30	1172.72	3469.60	2669.92	2669.92	1309.06
133	1209.45	1187.18	1187.23	1178.69	53.90	58.66	59.37	431.65
135	1238.43	1192.25	1192.25	1198.76	1869.90	1964.85	1964.85	3250.88
138	1260.18	1242.38	1242.38	1244.23	556.63	532.44	532.44	2626.04
139	1272.72	1248.91	1248.91	1245.24	14623.65	12182.49	12182.49	4042.38
140[a]	1305.79			1245.47	0.00			315206.78
141	1305.81	1225.46	1225.46	1243.56	109.43	117.15	117.15	735.89
143	1326.46	1268.40	1268.40	1282.99	39.89	35.28	35.29	258.82
147	1351.81	1312.57	1312.57	1315.01	3527.87	3125.65	3125.65	306.80
155	1386.76	1296.82	1296.82	1319.85	27.04	9.70	9.71	72.53
157	1392.85	1300.02	1300.00	1323.70	199.87	41.15	40.66	447.16
160	1402.29	1368.25	1368.25	1368.11	29.36	*	*	124727.40
163	1415.77	1371.10	1371.09	1384.35	420.77	590.77	590.89	676.56
164[b]	1417.37			1374.41	0.00			29002.79
165	1419.90	1386.83	1386.88	1385.24	722.39	1092.32	1076.60	2241.41
169	1429.70	1339.34	1339.16	1357.23	2001.52	1357.80	1341.93	53.80
171	1457.80	1421.40	1421.63	1412.56	2214.77	1637.81	1695.60	640.75
185	1498.59	1456.39	1456.39	1454.59	56.59	155.19	155.19	807.07
189	1506.30	1465.19	1465.19	1461.87	96.30	103.53	103.52	161.34
192	1515.04	1492.69	1492.68	1496.43	700.16	373.73	374.39	50.49
195	1550.37	1510.06	1510.06	1530.40	1568.46	1003.28	1003.90	10905.82
197	1554.65	1515.60	1515.60	1514.76	1121.04	1181.17	1180.55	12029.66
198	1560.16	1519.13	1519.13	1511.08	30.85	30.02	30.32	1509.02

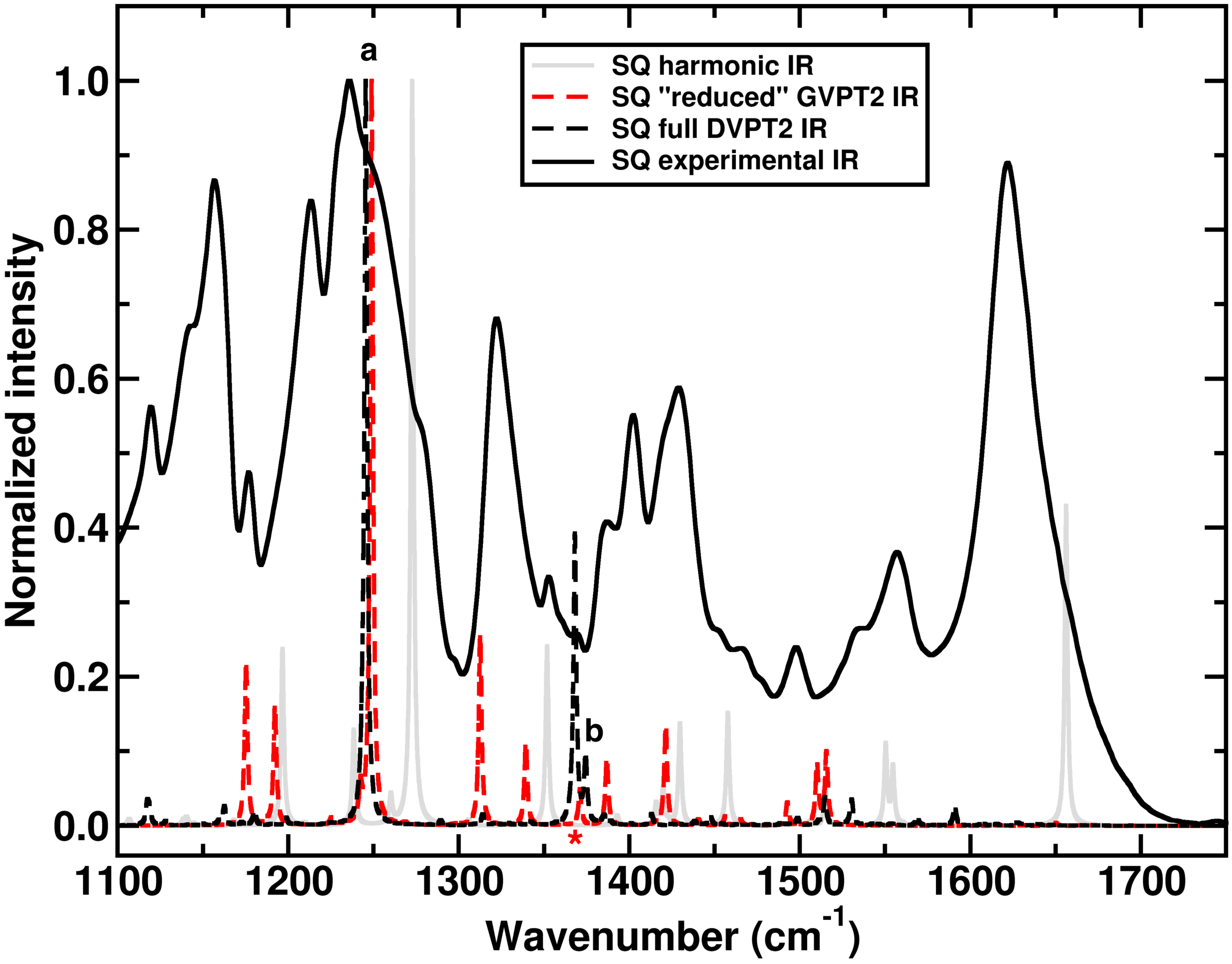

Figure S10: **SQ** B3LYP/6−31+G(d,p)/C−PCM acetonitrile harmonic (solid gray), selected *active* normal modes GVPT2 (dashed red), full DVPT2 (solid black) and experimental solid state (solid black) IR spectra in the $1100 - 1750\,\mathrm{cm}^{-1}$ wavenumber range (spectral region **b**). Computed intensities were uniformly scaled to obtain the most intense band to unity for a better comparison. Please refer to Table S1 for mode selection and for the divergent anharmonic intensities. Please refer to Table S3 for the comparison of the several used anharmonic models and Table S4 for the mean intensities and frequencies differences between the several anharmonic models. The latin letters indicate the modes that are particularly intense under the deployment of the full DVPT2 treatment which were not included in the selection of the *active* normal modes.

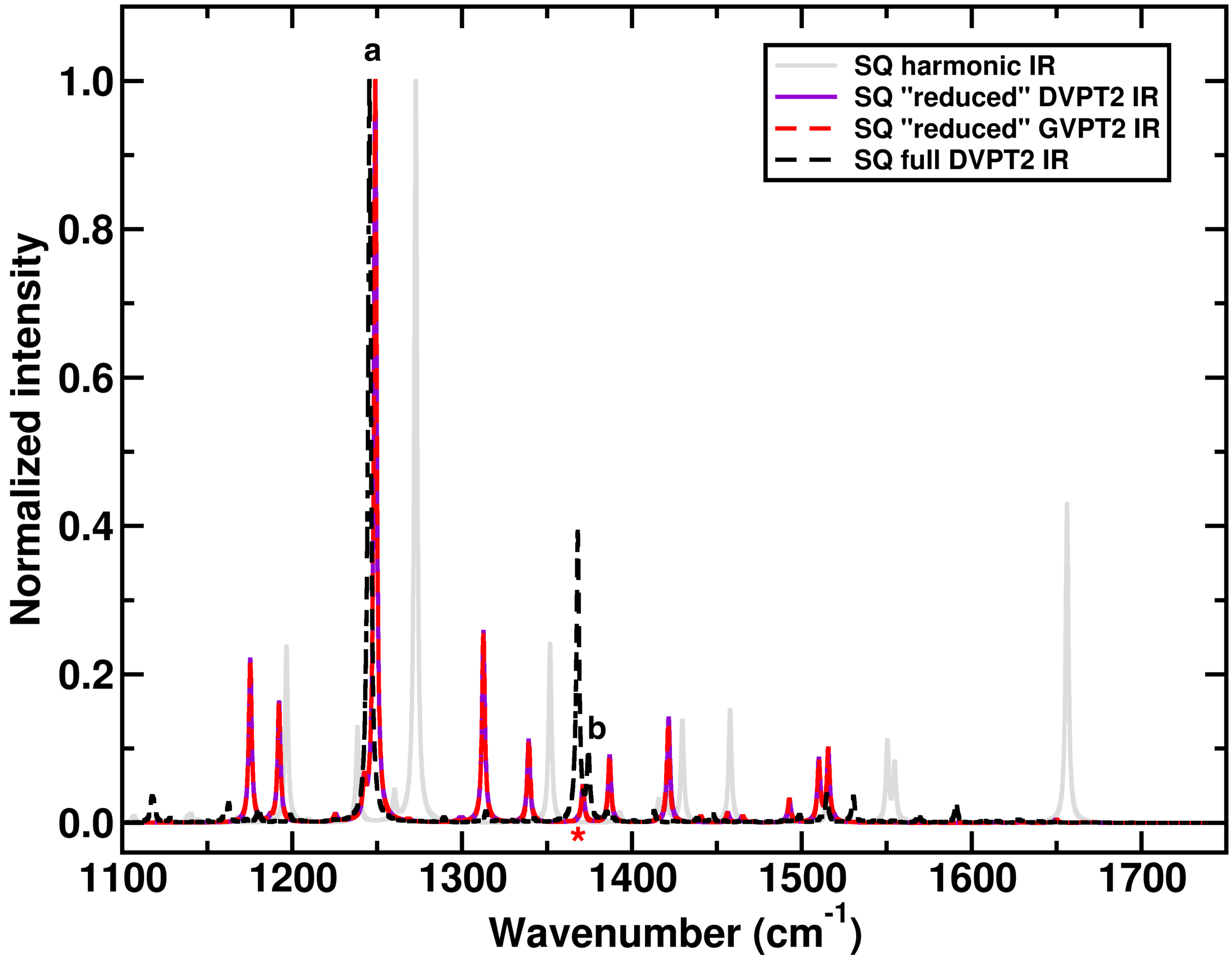

Figure S11: **SQ** B3LYP/6−31+G(d,p)/C−PCM acetonitrile harmonic (solid gray), selected *active* normal modes DVPT2 (solid violet), selected *active* normal modes GVPT2 (dashed red) and full DVPT2 (dashed black) IR spectra in the $1100 - 1750\,\mathrm{cm}^{-1}$ wavenumber range (spectral region **b**). Computed intensities were uniformly scaled to obtain the most intense band to unity for a better comparison. Please refer to Table S1 for mode selection and for the divergent anharmonic intensities. Please refer to Table S3 for the comparison of the several used anharmonic models and Table S4 for the mean intensities and frequencies differences between the several anharmonic models. The latin letters indicate the modes that are particularly intense under the deployment of the full DVPT2 treatment which were not included in the selection of the *active* normal modes.

Table S4: B3LYP/6−31+G(d,p)/C−PCM acetonitrile **SQ** IR mean frequency and intensity difference evaluations of several anharmonic approaches with respect to the harmonic (top) and "reduced" GVPT2 (bottom) reference values in the **b** spectral region. Please refer to Section 1 for how we evaluated the mean differences. See Tables S1 and S3 for modes selection and the comparison between the harmonic and the anharmonic approaches. Mean frequency difference is expressed as cm^{-1} and mean intensity difference as %.

	Frequency [cm^{-1}]	Intensity [%]
GVPT2r vs. harmonic	~ -46	$\sim +13$
DVPT2r vs. harmonic	~ -46	$\sim +13$
DVPT2f vs. harmonic	~ -42	~ -21
DVPT2r vs. GVPT2r	~ 0	~ 0
DVPT2f vs. GVPT2r	$\sim +5$	~ -38

8 Anharmonic–Computed Infrared and Non–Resonant Raman Fundamental Modes of DPSQ

Table S5: B3LYP/6−31+G(d,p)/C−PCM harmonic and selected *active* anharmonic fundamental infrared modes of **DPSQ** in acetonitrile. In parenthesis, computed infrared intensities are expressed as km/mol. If the anharmonic intensity diverges, we labeled it with an asterisk and we used the intensity of the corresponding harmonic calculation in plotting the spectra in Figure S9, dashed blue line.

Mode	Harmonic [cm^{-1}, km/mol]	Anharmonic [cm^{-1}, km/mol]
140	1189.64 (1618.29)	1168.23 (1498.65)
141	1193.92 (1.03)	1178.72 (18.91)
146	1243.62 (428.72)	1211.26 (456.03)
148	1270.26 (4968.76)	1244.10 (13320.87)
149	1271.51 (21851.55)	1249.58 (12293.47)
150	1282.64 (545.46)	1256.12 (503.81)
153	1327.36 (61.48)	1302.02 ($*$)
155	1339.73 (505.21)	1313.81 (289.77)
163	1395.94 (952.41)	1343.57 (414.31)
165	1416.29 (590.03)	1365.09 (444.96)
167	1451.49 (3454.74)	1411.31 (2910.30)
168	1482.28 (45.73)	1458.32 (40.56)
173	1512.15 ($1.1 \cdot 10^{-3}$)	1479.45 ($7.8 \cdot 10^{-3}$)
174	1515.59 (735.72)	1481.29 (456.68)
175	1522.50 (85.24)	1483.30 (31.37)
176	1523.89 (103.63)	1491.00 ($*$)
180	1550.85 (47,53)	1506.73 (1042.26)
182	1559.00 (2732.62)	1522.82 (2182.199)
185	1626.46 (29.70)	1588.42 ($*$)
186	1628.94 (832.35)	1592.60 (1233.85)

Table S6: B3LYP/6−31+G(d,p)/C−PCM harmonic and selected *active* anharmonic fundamental non–Resonant Raman modes of **DPSQ** in acetonitrile. In parenthesis, computed Raman activity is expressed as $Å^6$. If the anharmonic intensity diverges, we labeled it with an asterisk and we used the intensity of the corresponding harmonic calculation in plotting the spectra in Figure S9, dashed red line.

Mode	Harmonic $[cm^{-1}, (Å^6)]$	Anharmonic $[cm^{-1}, (Å^6)]$
19	140.72 (33.26)	145.41 (35.38)
20	167.26 (1.41)	165.16 (1.57)
22	192.35 (8.84)	194.15 (9.05)
26	228.59 (6.51)	227.27 (6.74)
28	237.93 (2.81)	227.65 (2.66)
29	259.88 (46.68)	248.81 (39.20)
30	259.94 (0.02)	249.77 (3.24)
41	419.63 (6.52)	404.50 (7.20)
42	419.66 $(2.4 \cdot 10^{-3})$	404.61 $(1.1 \cdot 10^{-3})$
43	420.20 $(8.4 \cdot 10^{-5})$	406.78 (58.62)
44	420.42 (72.10)	408.07 (70.06)
57	577.46 (75.28)	574.22 (66.74)
58	591.51 (0.28)	585.34 (0.23)
151	1282.80 (70.90)	1257.01 (62.95)
152	1327.34 (8.24)	1301.88 (∗)
154	1338.43 (0.37)	1312.14 (1.08)
160	1359.60 (45.82)	1318.76 (10.52)
162	1365.98 (15.40)	1330.32 (12.68)
177	1523.94 (13.17)	1491.35 (∗)
178	1526.12 (0.01)	1494.87 (599.86)
179	1526.23 (61.26)	1492.43 (23.46)
181	1553.88 (7.39)	1511.59 (6.01)

Mode	Proposed mode assignment	Harmonic [cm^{-1}, (Å^6)]	Anharmonic [cm^{-1}, (Å^6)]	Experimental
19	β N,N'-disubstituents	140.72 (33.26)	145.41 (35.38)	142
44	τ N,N'-disubstituents	420.42 (72.10)	408.07 (70.06)	441
57	τ phenolic rings	577.46 (75.28)	574.22 (66.74)	572
151	δ C−N N,N'-disubstituents	1282.80 (70.90)	1257.01 (62.95)	1249
160	δ O−H iHB	1359.60 (45.82)	1318.76 (10.52)	1394
177		1523.94 (13.17)	1491.35 (∗)	
178	phenyl δ −CH N,N'-disubstituents	1526.12 (0.01)	1494.87 (599.86)	1540
179		1526.23 (61.26)	1492.43 (23.46)	

Table S7: B3LYP/6−31+G(d,p)/C−PCM harmonic and selected *active* GVPT2 anharmonic non–Resonant Raman fundamental modes compared with experimental findings for **DPSQ** in acetonitrile. In parenthesis, computed Raman activity is expressed as Å^6. If the anharmonic Raman activity diverges, we report the intensity of the corresponding harmonic calculation and we labeled it with an asterisk. Raman mode assignment symbols and abbreviations: β−breathing; iHB−intramolecular hydrogen bond moiety; ρ−rocking; ν−stretching; δ−scissoring; τ−twisting. The visualization of the selected modes are reported in Figures 6 and 7.

9 Anharmonic Infrared and Non–Resonant Raman Fundamental Modes of DBSQ

Table S8: B3LYP/6−31+G(d,p)/C−PCM harmonic and selected *active* anharmonic fundamental infrared modes of **DBSQ** in acetonitrile. In parenthesis, computed infrared intensities are expressed as km/mol.

Mode	Harmonic [cm^{-1}, km/mol]	Anharmonic [cm^{-1}, km/mol]
154	1189.19 (2212.91)	1168.23 (934.13)
155	1199.51 (583.78)	1174.80 (695.87)
160	1203.57 (238.88)	1181.78 (1514.20)
162	1216.02 (39.94)	1198.05 (47.01)
163	1225.71 (2313.87)	1203.67 (895.72)
166	1258.95 (605.52)	1231.78 (94.01)
167	1269.00 (14183.61)	1255.07 (31.06)
168	1303.83 (105.39)	1267.09 (100.13)
171	1322.71 (103.89)	1287.70 (3.86)
173	1322.77 (99.85)	1283.53 (13.22)
175	1357.34 (1590.96)	1323.38 (110.38)
179	1362.43 (1486.87)	1334.87 (315.68)
183	1387.27 (142.90)	1356.06 (35.62)
185	1393.96 (293.62)	1357.91 (20.70)
187	1420.77 (514.44)	1372.75 (11.44)
189	1427.31 (3143.37)	1389.84 (24.31)
191	1460.28 (1729.35)	1424.05 (41.38)
193	1482.49 (26.83)	1458.23 (40.00)
197	1486.48 (92.27)	1447.83 (24.58)
199	1507.11 (24.44)	1465.89 (0.09)
201	1516.94 (697.14)	1488.72 (264.71)
204	1529.91 (80.90)	1498.19 (1322.12)
207	1552.43 (310.16)	1515.38 (59.54)
209	1557.32 (2404.95)	1522.71 (830.82)

Table S9: B3LYP/6−31+G(d,p)/C−PCM harmonic and selected *active* GVPT2 anharmonic fundamental non–resonant Raman modes of **DBSQ** in acetonitrile. In parenthesis, computed Raman activity is expressed as $Å^6$.

Mode	Harmonic $[cm^{-1}, (Å^6)]$	Anharmonic $[cm^{-1}, (Å^6)]$
19	120.86 (6.02)	120.94 (9.49)
20	130.98 $(3.9 \cdot 10^{-5})$	124.09 $(2.0 \cdot 10^{-4})$
21	135.48 (3.58)	110.18 (4.39)
22	147.58 (10.65)	129.14 (8.76)
25	170.99 (5.49)	156.39 (5.69)
45	385.91 (8.76)	374.49 (7.32)
48	415.13 (0.87)	391.65 (0.06)
49	415.18 (0.02)	394.45 (0.03)
50	415.23 $(7.7 \cdot 10^{-5})$	393.20 (1.20)
52	461.82 (13.53)	451.09 (14.39)
65	580.48 (32.04)	577.39 (31.35)
194	1483.25 (1.59)	1460.63 (1.44)
195	1484.41 $(3.4 \cdot 10^{-6})$	1461.05 $(2.7 \cdot 10^{-3})$
196	1486.32 (1.79)	1446.06 (1.72)
197	1486.48 (0.01)	1444.99 (2.63)
198	1500.50 (35.00)	1460.54 (28.19)
199	1507.11 $(2.2 \cdot 10^{-5})$	1465.59 $(2.1 \cdot 10^{-3})$
200	1514.20 (0.20)	1488.58 (0.22)
202	1527.74 (6.47)	1494.95 (4.75)

Mode	Proposed mode assignment	Harmonic [cm^{-1}, (Å^6)]	Anharmonic [cm^{-1}, (Å^6)]	Experimental (cm^{-1})
22	β N,N'-disubstituents	147.58 (10.65)	129.14 (8.76)	146
52	β,τ $-CH_2$ N,N'-disubstituents	461.82 (13.53)	451.09 (14.39)	464
65	τ phenolic rings	580.48 (32.04)	577.39 (31.35)	575
198	δ O$-$H iHB, $-CH_2$, phenolic C$-$H	1500.50 (35.00)	1460.54 (28.19)	1491<br>1497
202	δ O$-$H iHB, $-CH_2$ N,N'-disubstituents	1527.74 (6.47)	1494.95 (4.75)	1497

Table S10: B3LYP/6$-$31+G(d,p)/C$-$PCM acetonitrile harmonic and selected *active* anharmonic non–Resonant Raman fundamental modes compared with experimental findings for **DBSQ**. In parenthesis, computed Raman activity is expressed as Å^6. Raman mode assignment symbols and abbreviations: $\beta-$breathing; iHB$-$intramolecular hydrogen bond moiety; $\rho-$rocking; $\nu-$stretching; $\delta-$scissoring; $\tau-$twisting. The visualization of the selected modes are reported in Figure S12.

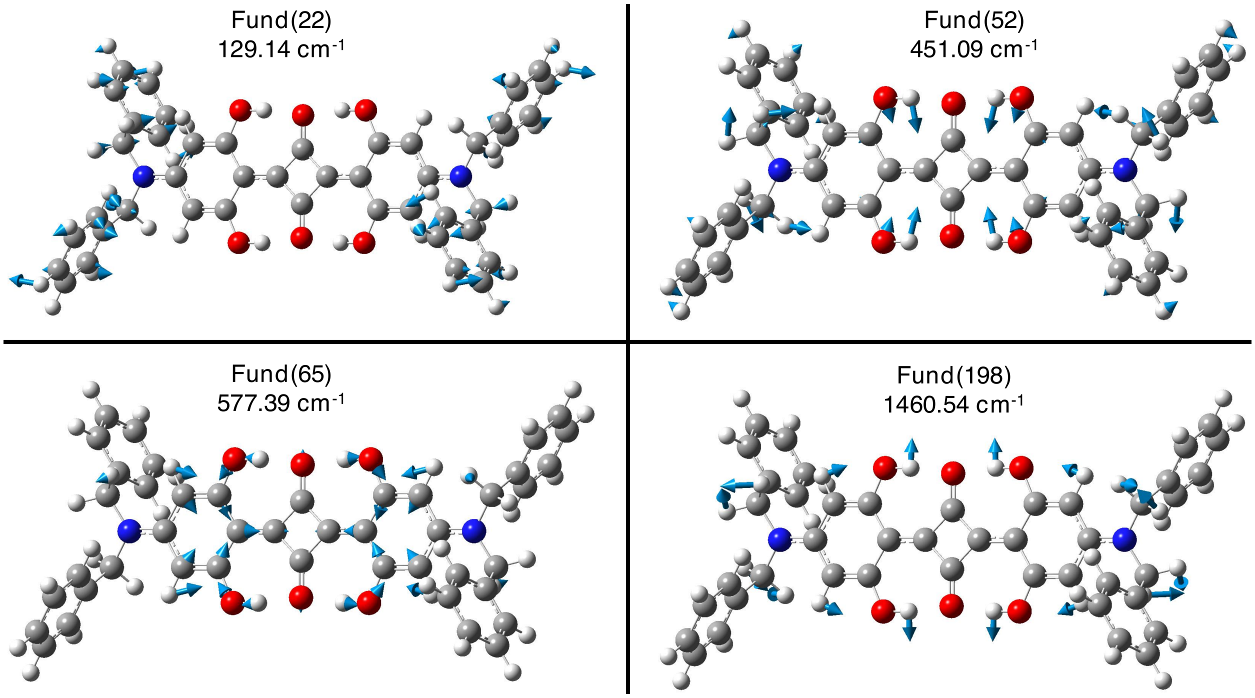

Figure S12: Visualization of B3LYP/6−31+G(d,p)/C−PCM non–Resonant Raman active fundamental low– and higher–intensities modes of **DBSQ** in acetonitrile that better match with experimental observations (please refer to Table S10) with their displacement vectors and anharmonic values. Atoms color palette: C-dark gray; H-white; N-blue; and O-red.

References

(1) Buttarazzi, E.; Inchingolo, A.; Pedron, D.; Alberto, M. E.; Collini, E.; Petrone, A. Conformational and environmental effects on the electronic and vibrational properties of dyes for solar cell devices. *J. Chem. Phys.* **2024**, *160*, 204301.
